# Cholinergic modulation in the vertebrate auditory pathway

**DOI:** 10.3389/fncel.2024.1414484

**Published:** 2024-06-19

**Authors:** Chao Zhang, R. Michael Burger

**Affiliations:** ^1^Cold Spring Harbor Laboratory, Cold Spring Harbor, NY, United States; ^2^Department of Biological Sciences, Lehigh University, Bethlehem, PA, United States

**Keywords:** acetylcholine, modulation, auditory, sound, nicotinic, muscarinic

## Abstract

Acetylcholine (ACh) is a prevalent neurotransmitter throughout the nervous system. In the brain, ACh is widely regarded as a potent neuromodulator. In neurons, ACh signals are conferred through a variety of receptors that influence a broad range of neurophysiological phenomena such as transmitter release or membrane excitability. In sensory circuitry, ACh modifies neural responses to stimuli and coordinates the activity of neurons across multiple levels of processing. These factors enable individual neurons or entire circuits to rapidly adapt to the dynamics of complex sensory stimuli, underscoring an essential role for ACh in sensory processing. In the auditory system, histological evidence shows that acetylcholine receptors (AChRs) are expressed at virtually every level of the ascending auditory pathway. Despite its apparent ubiquity in auditory circuitry, investigation of the roles of this cholinergic network has been mainly focused on the inner ear or forebrain structures, while less attention has been directed at regions between the cochlear nuclei and midbrain. In this review, we highlight what is known about cholinergic function throughout the auditory system from the ear to the cortex, but with a particular emphasis on brainstem and midbrain auditory centers. We will focus on receptor expression, mechanisms of modulation, and the functional implications of ACh for sound processing, with the broad goal of providing an overview of a newly emerging view of impactful cholinergic modulation throughout the auditory pathway.

## Introduction

**Figure 1:**
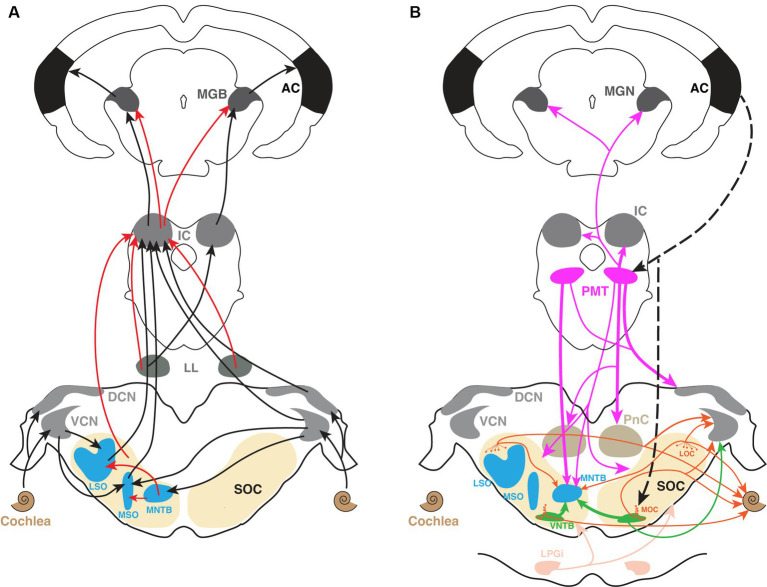
The mammalian ascending auditory pathway and its interactions with a complex cholinergic network. **(A)** The major components of the ascending auditory pathway from cochlea to the auditory cortex. *Black arrows* indicate excitation while *red arrows* indicate inhibition. The superior olive and its constituent nuclei are shown in *beige* and *blue*, respectively. **(B)** A summary of the known cholinergic connectivity within the auditory system. The colors of the arrows match the colors of the different cholinergic sources. Thickness of the arrows from the same source indicates the relative strength of cholinergic outputs based on anatomical descriptions. AC, auditory cortex; DCN, dorsal cochlear nucleus; IC, inferior colliculus; LL, lateral lemniscus; LOC, lateral olivecochlear; LPGi, lateral paragigantocellular nucleus; LSO, lateral superior olive; MGN, medial geniculate nucleus; MNTB, medial nucleus of trapezoid body; MOC, medial olivecochlear; MSO, medial superior olive; PMT, pontomesencephalic tegmentum; PnC, pontine reticular nucleus; SOC, superior olivary complex; VCN, ventral cochlear nucleus; VNTB, ventral nucleus of trapezoid body.

The vast richness of sound sensation endows us with the ability to communicate, enjoy music, and navigate the world. These auditory experiences rely on an impossibly complex neural architecture that spans every major division of the central nervous system. Despite decades of research investigating the myriad components of this pathway, fundamental discoveries regarding its basic organizational and functional features continue to emerge with astonishing frequency. In recent years, new evidence has shown that modulatory circuitry is overlaid upon, and integrated within these circuits, adding further complexity to the fundamental neural computations that enable hearing in animals. This review focuses on recent work investigating cholinergic modulation along this pathway. These studies demonstrate that cholinergic circuits appear to influence auditory function at all levels and in a diversity of ways, from sound transduction in the inner ear to the auditory cortex (Figure 1).

## General organization of the ascending auditory pathway

Sound is first transduced into electrical signals in the hair cells of the inner ear before processing through a rich and interconnected network of auditory centers from the ear to cortex in vertebrates. Afferent auditory nerve fibers encode and relay this information to several distinct neuron types in the cochlear nucleus (CN) of the brain (Osen, 1969a, 1970; Young and Brownell, 1976; Rhode et al., 1983; Oertel et al., 2002; Maclaine and Llano, 2020). The mammalian cochlear nucleus complex is composed of two major divisions, the ventral (VCN) and dorsal cochlear nuclei (DCN) (Osen, 1969b). The VCN receives primary excitatory innervation from the inner ear via auditory nerve, therefore serving as the entry point of acoustic information into the brain (Arnesen and Osen, 1978; Oertel et al., 1990). The DCN is a cerebellum-like structure that receives both auditory nerve input as well as input from VCN and other brain regions (Osen, 1969b; Moore and Osen, 1979; Hackney et al., 1990; Oertel and Cao, 2020). The major outputs from the CN branch to form terminals in both ipsilateral and contralateral brainstem nuclei as well as the contralateral midbrain (Harrison and Irving, 1966; Bruce Warr, 1995; Davis, 2005). In the brainstem, the superior olivary complex (SOC) receives bilateral input from both CNs, and performs numerous fundamental auditory computations including those related to sound localization (Stotler, 1953; Kiss and Majorossy, 1983; Glendenning et al., 1985, 1991; Thompson and Schofield, 2000). The SOC is composed of both primary SOC nuclei and periolivary nuclei. The primary SOC comprises the medial nucleus of trapezoid body (MNTB), medial superior olive (MSO) and lateral superior olive (LSO). These nuclei have been intensively studied for their physiological properties as major centers of binaural computations for sound-localization (Goldberg and Brown, 1969; Yin and Chan, 1990; Grothe and Sanes, 1993; Pollak et al., 2003; Burger and Rubel, 2008; Grothe and Pecka, 2014; Risoud et al., 2018). SOC nuclei output to lateral lemniscus (LL) ipsilaterally and inferior colliculus (IC) bilaterally (Adams, 1979; Glendenning et al., 1981; Schofield, 1991; Oliver et al., 1995; Malmierca and Merchán, 2004; Henkel, 2018). Lemniscal nuclei in turn project to both ipsilateral and contralateral inferior colliculus (IC) in the midbrain (Harrison and Howe, 1974; Schofield, 2002). In this way, the IC is a major integration center of auditory circuitry, serving as the point of convergence for nearly all afferent pathways emanating from lower nuclei (Pollak et al., 1986; Zook and Casseday, 1987; Ito et al., 2016; Maclaine and Llano, 2020). The IC projects to the ipsilateral thalamic medial geniculate nucleus (MGN) (Tachibana et al., 1979; Kudo and Niimi, 1980; Rouiller and de Ribaupierre, 1985; Mellott et al., 2019), from which the acoustic information is then conveyed to the primary auditory cortex (AC) (Oliver and Hall, 1978; Brugge and Howard, 2002). Two subdivisions of AC, the primary and secondary AC (A1, A2, respectively) receive auditory input from MGN, thus completing the major afferent circuitry (Strutz, 1987; Bizley, 2017).

## A pervasive cholinergic network influences the ascending auditory pathway

ACh was first chemically identified by Baeyer (1867). Its recognition as a neurotransmitter by Dale (1914) and Loewi and Navratil (1926), initiated over a century of studies on this biochemically important molecule. Upon released into the synaptic cleft, ACh binds to one of many ionotropic or metabotropic acetylcholine receptors (AChRs) that influences the physiology of neurons is a wide variety of ways. Cholinergic function has been extensively studied in high ordered auditory neurons, like those of the thalamus and cortex. For example, the forebrain nucleus basalis (NB) serves as the main cholinergic source projecting widely to neocortex. Using *in vivo* recording, Froemke et al. (2007) discovered that NB-originating cholinergic input is capable of shaping the frequency tuning of auditory cortex (A1) neurons, by simultaneously increasing excitatory input and dampening inhibitory input. In a complimentary study, Leach et al. (2013) conducted behavioral studies on ferrets and discovered that loss of NB cholinergic circuitry reduces the accuracy of localizing brief sounds, and prevents adaptation to chronic occlusion of one ear, thereby significantly impairing sound-localization ability. ACh has also been suggested to modulate several important AC computational functions including spatial receptive fields (Froemke et al., 2007; Metherate, 2011), frequency selectivity (Ashe et al., 1989; McKenna et al., 1989; Metherate and Weinberger, 1989, 1990), tuning curves (Froemke et al., 2007; Metherate et al., 2012), rate-level functions (RLF) (McKenna et al., 1988; Metherate et al., 1990; Metherate and Weinberger, 1990; Kawai et al., 2007), sound-evoked firing patterns (Metherate et al., 1992), intra-cortical communication (Froemke et al., 2007; James et al., 2019) and cognitive function (Metherate, 2004; Liang et al., 2006, 2008; Leach et al., 2013). In the thalamus, ACh has been suggested to influence the firing pattern and encoding efficacy of MGN neurons (Mooney et al., 2004; Varela and Sherman, 2007; Hamada et al., 2010; Sottile et al., 2017; Richardson et al., 2021). Overall, the cholinergic modulation on AC and auditory thalamus strongly suggested the critical role of ACh in mediating higher-ordered sensory processing. For a thorough review of this work see (Metherate and Hsieh, 2004; Richardson et al., 2021; Kunnath et al., 2023).

More recently, work in the auditory brainstem has extended this general view of cholinergic modulation to lower ordered central processing. Studies on cholinergic function by our lab and others reveals that ACh makes major contributions to fundamental neural computations that enable or enhance features such as signal detection in noise and sound intensity encoding in several brainstem and midbrain regions. Here we will describe findings from our recent studies and those of other laboratories on cholinergic modulation that are building a new appreciation of its pervasive and complex functionality to light.

### A diversity of acetylcholine receptors mediate intrinsic properties of neurons

**Figure 2:**
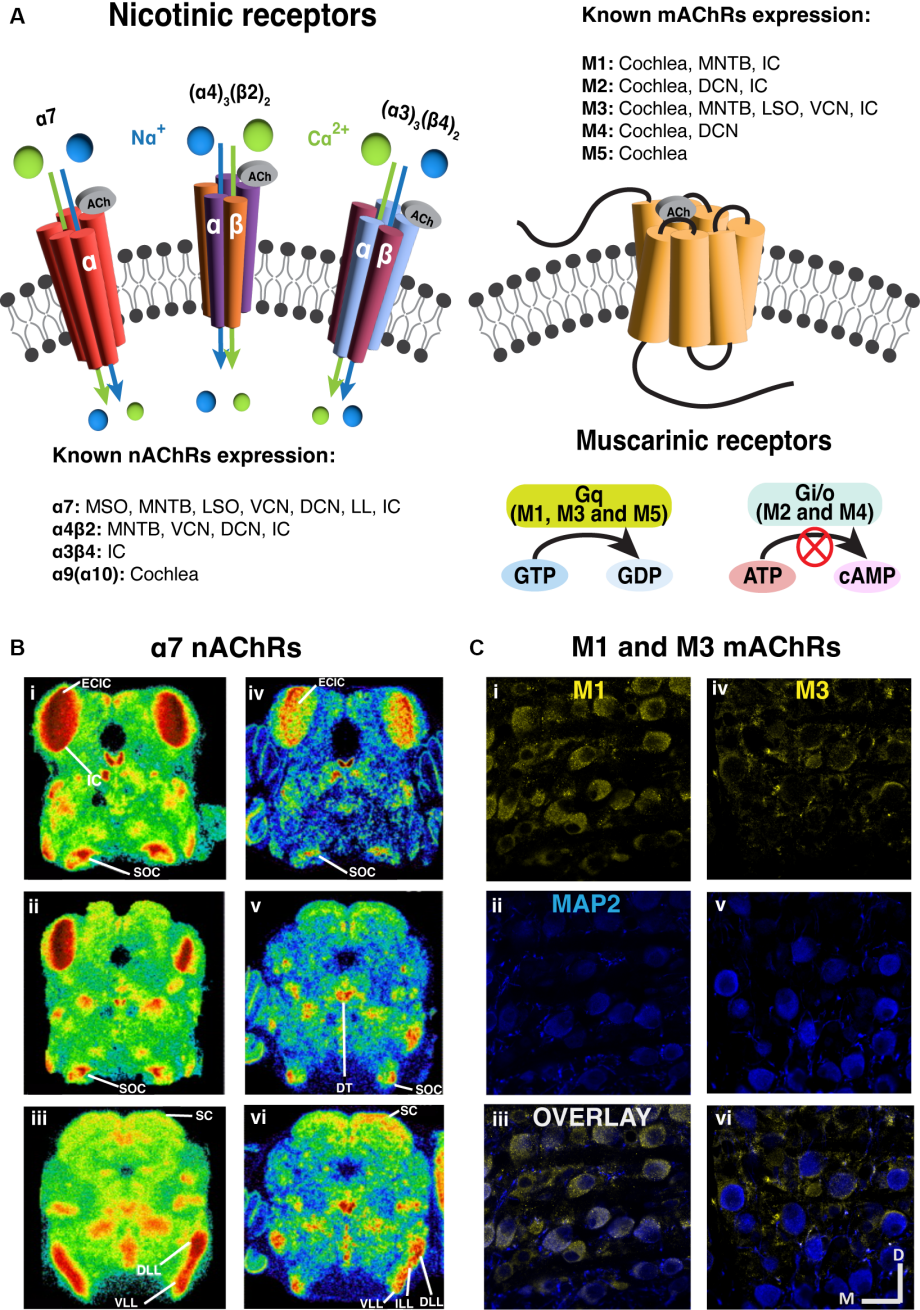
Prevalent expression of nicotinic and muscarinic acetylcholine receptors along the brainstem auditory pathway. **(A)** The structure and signaling pathways of ionotropic nicotinic and metabotropic muscarinic cholinergic receptors in the CNS. Three representative nAChRs, α7, (α4)3(β2)2 and (α3)3(β4)2 subtypes are shown, showing that most nAChRs are ligand gated ion channels. On the other hand, most mAChRs are G protein-coupled receptors (GPCR). M1, M3 and M5 subtypes are Gq-coupling excitatory receptors, while M2 and M4 are Gi/o-coupling inhibitory receptors. **(B)** The expression of α7 nAChRs are prevalent in the auditory brainstem [adapted from Happe and Morley (2004), with permission from Elsevier; license # 5762610422615]. Left panels **(i–iii)**: Representative images of mouse α7 mRNA *in situ* hybridization at several levels of the auditory brainstem at P10. Right panels **(iv–vi)**: Representative images of rat 125I-α-bungarotoxin binding at several levels of the auditory brainstem at P10. **(C)** Immunohistochemical labeling shows that both M1 and M3 mAChRs expression in the gerbil medial nucleus of trapezoid body [MNTB, adapted from Weimann et al. (2024), with permission from the Journal of Neuroscience under CC-BY license]. M1 expression yellow label **(i–iii)** appears to be somatic while M3 labeling yellow label **(iv–vi)** suggests presynaptic localization. The microtubule associated protein 2 (MAP2) was labeled in blue. DCN, dorsal cochlear nucleus; DLL, dorsal lateral lemniscus; DT, dorsal tegmental; ECIC, external cortex of inferior colliculus; IC, inferior colliculus; ILL, intermediate lateral lemniscus; LL, lateral lemniscus; LSO, lateral superior olive; MNTB, medial nucleus of trapezoid body; MOC, medial olivecochlear; MSO, medial superior olive; SC, superior colliculus; SOC, superior olivary complex; VCN, ventral cochlear nucleus; VLL, ventral lateral lemniscus.

There are two broad categories of AChRs, muscarinic and nicotinic, named for the agonistic effects of the fungal toxin muscarine or the plant toxin nicotine on the receptors (Servent et al., 2011). Muscarinic receptors (mAChRs) comprise a diverse class of metabotropic G-protein coupled receptors (GPCRs) (Ishii and Kurachi, 2006). Among the five major subtypes of mAChR, referred to as M1-M5; M1, M3 and M5 subtypes engage Gq proteins and trigger IP3 and calcium signaling pathways upon receptor activation (Roeren et al., 1988; Caulfield and Birdsall, 1998; Eglen, 2006). In contrast, M2 and M4 subtypes employ Gi proteins to down-regulate adenylyl cyclase, and subsequently decrease protein kinase A activity (Dell'Acqua et al., 1993). Through both pathways, mAChRs can function to indirectly influence the activity of ion channels in the cytoplasmic membrane and influence neural signaling. Typically, M1, M3, and M5 receptor activation imparts a net excitatory effect, while M2 and M4 activation drives inhibitory/suppressive effects (Figure 2).

Globally, the excitatory mAChRs effects derive from suppressive modulation of potassium channels (Brown, 2018). These potassium channels include voltage dependent Kv7 (KCNQ) (Brown and Adams, 1980; Womble and Moises, 1992), leak K2p (KCNP) (Madison et al., 1987; Womble and Moises, 1992; Coggan et al., 1994), G-protein-coupled inward rectifiers (GIRK) (Uchimura and North, 1990), and Ca^++^ activated potassium SK family channels that are responsible for the slow afterhyperpolarization current I_AHP_ (Madison et al., 1987; Coggan et al., 1994). mAChR-dependent block of K_v_7-mediated outward M-current (I_M_) enhances excitability by producing membrane depolarization, increasing input resistance and reducing action potential threshold. In the auditory system, the Kv7 expression has been documented in several regions. Anatomical evidence suggested the prevalence of Kv7.5 in synaptic endings of the rat auditory brainstem nuclei, including CN, LSO, MSO, SPN, MNTB, LL, and IC (Caminos et al., 2007; Garcia-Pino et al., 2010). Kv7.2, 7.3 and 7.4 were anatomically identified in mammalian IC (Wang et al., 1998; Kharkovets et al., 2000). Kv7.4 was also found in cochlear hair cells, CN and LL (Kharkovets et al., 2000). The presence of Kv7 in these regions strongly suggests the co-expression of mAChRs. Other than Kv7-mediated modulation of excitation, blockage of voltage-insensitive leak K^+^ channels by muscarinic receptors was observed to excite T stellate cells of VCN (Fujino and Oertel, 2001). On the other hand, muscarinic receptors have also been shown to hyperpolarize neurons and thereby decrease neuron excitability, by activating GIRK channels in Golgi cells of DCN (Irie et al., 2006) and in caudal pontine reticular nucleus (Bosch and Schmid, 2006). In addition, post-synaptic M1/M3 mAChRs activation has been shown to influence synaptic plasticity by converting long-term potentiation (LTP) into long-term depression (LTD) in mouse principal DCN neurons (Zhao and Tzounopoulos, 2011). This effect is likely due to an interplay between the cholinergic system and another potent modulator, the endocannabinoid system. It is suggested that upon activation of these mAChRs, the postsynaptic GPCR-coupled intracellular cascade enhanced modulation by parallel endocannabinoid signaling impinging on the same DCN neuron.

Nicotinic receptors (nAChRs) are ionotropic cation channels (Itier and Bertrand, 2001; Hammond, 2015) composed of 5 subunits. These channels are variably selective for Na^+^, K^+^ and Ca^2+^ depending on subunit composition (Beker et al., 2003; Weber et al., 2005), where alpha-7 subunit containing channels exhibit prominent Ca^2+^ permeability. The influx of Ca^2+^ through alpha-7 subunit containing receptors has been shown to increase neurotransmitter release when these receptors are expressed in pre-synaptic terminals (Shen and Yakel, 2009; Uteshev, 2012). Generally, when ionotropic nAChRs are excitatory and expressed post-synaptically, their activation leads to cation influx and depolarization (Changeux and Paas, 2009; Akers et al., 2020).

### Cholinergic neurons are both extrinsic and intrinsic to the auditory system

The complexity of afferent auditory circuitry is complemented by a similarly complex network of cholinergic circuitry throughout the system. Some of these cholinergic projections arise from cholinergic or neuromodulatory nuclei outside of the canonical auditory centers, while other cholinergic neurons are intrinsic to the auditory pathway itself. Sources of ACh that project to AC have been identified primarily in the basal forebrain, while several regions between the cochlear nucleus and thalamus receive major cholinergic projections from the midbrain pontomesencephalic tegmentum (PMT) (Shute and Lewis, 1967; Hallanger et al., 1987; Schofield et al., 2011; Figure 1B). Furthermore, cholinergic cells have been identified in the SOC of rats (Sherriff and Henderson, 1994), guinea pigs (Motts et al., 2008), gerbils (Beebe et al., 2021; Zhang et al., 2021), mouse (Beebe et al., 2023) and human (Mizukawa et al., 1986; Heckers et al., 1992). Among the periolivary nuclei, the ventral nucleus of trapezoid body (VNTB) has been identified as a major cholinergic source to CN in rats (Gomez-Nieto et al., 2008), guinea pigs (Mellott et al., 2011), gerbils (Beebe et al., 2021), and mice (Beebe et al., 2023). Moreover, the lateral paragigantocellular nucleus (LPGi) has been recently identified as a novel cholinergic source that projects bilaterally to CN (Beebe et al., 2023) and SOC (Beebe et al., 2021). The LPGi has previously been associated with autonomic functions and sensory gating (Stornetta et al., 2013; Schofield and Hurley, 2018), and it has numerous connections with auditory structures (Andrezik et al., 1981; Kamiya et al., 1988). Additionally, neurons in VNTB and PMT have been shown to be driven by sound (Koyama et al., 1994; Reese et al., 1995a,b). Together these findings suggest that ACh release is triggered by numerous cholinergic sources upon sound presentation, and that this circuitry is poised to influence sound processing throughout the brain. Functionally, the involvement of a potent, sound-driven neuromodulator along the afferent pathway may render auditory neurons capable of dynamically adjusting excitability or synaptic transmission. These modulatory inputs maybe engaged to rapidly accommodate the demands of complex stimulus variations in intensity, spectral content, location and temporal structure. We will highlight some results that support this newly emerging broader view of cholinergic modulation.

## Cholinergic function is diverse along the ascending auditory pathway

### Cochlea and auditory nerve

**Figure 3:**
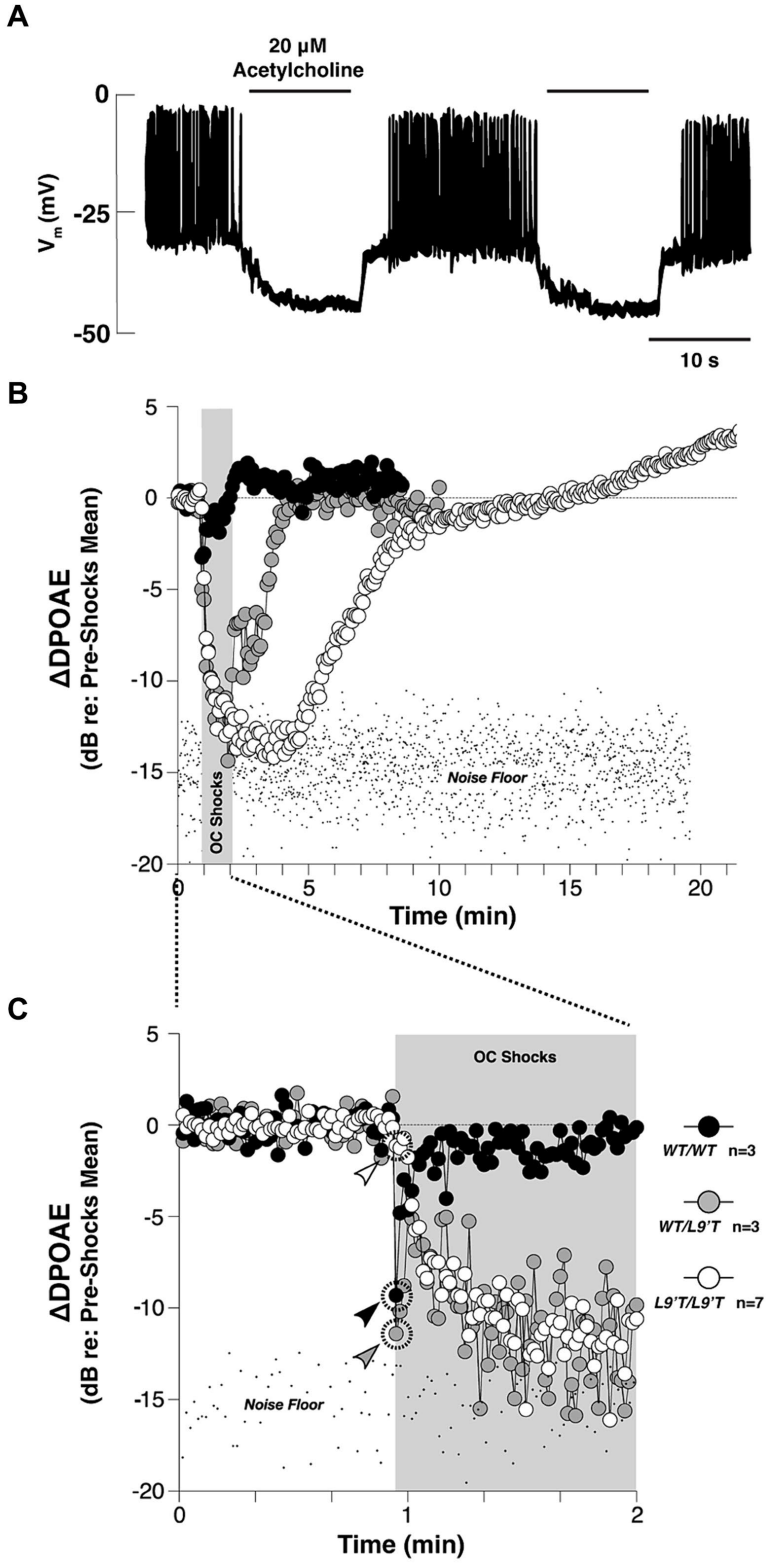
The cholinergic efferent feedback circuit suppresses hair cell activity and otoacoustic emissions from in the auditory periphery. **(A)** Cholinergic inhibition of Ca^2+^ action potentials in the rat inner hair cells (IHC) during whole-cell current clamp. Application of 20 μΜ ACh hyperpolarized the membrane potential by ~15 mV, and thereby abolished the generation of Ca^2+^ action potentials of the IHC in response to 120 pA injection current [Adapted from Glowatzki and Fuchs (2000); with permission from American Association for the Advancement of Science; license # 5752590513900]. **(B)** Olivocochlear (OC)-mediated suppression of cochlear distortion product otoacoustic emission (DPOAE) is mediated by α9-containing nAChRs [adapted from Taranda et al. (2009), with permission from PLOS Biology under CC-BY license]. The DPOAEs were measured from anesthetized mice before, during and after 70-s of shock trains delivered to the OC bundle indicated by the *gray bars*. The mutant nAChRs are more sensitive to ACh than the wild-type nAChRs. Compared to the wild-type, mutant mice showed slowed, enhanced and prolonged OC-mediated suppression. **(C)** The onset period of the OC train shown in **(B)**. Arrowheads in **(C)** indicate the first point after shock-train onset for each genotype. In both **(B,C)**, note that the suppression with hypersensitized nAChRs were so strong that the DPOAE amplitudes were driven below the background noise floor.

The auditory periphery includes a cochlear amplification function mediated by electromotile outer hair cells (OHC) that increases acoustic gain and enhances cochlear output over a range of stimulus intensities (Guinan, 1996; Darrow et al., 2006, 2007). Efferent cholinergic projections protect the auditory periphery from sound-induced damage via suppression of the cochlear amplification system (Wolpert et al., 2014; Guinan, 2018). Cholinergic olivo-cochlear neurons include medial olivo-cochlear (MOC) and lateral olivo-cochlear (LOC) neurons. The anatomy and physiology of these efferent circuits has been reviewed in detail (Ciuman, 2010; Künzel and Wagner, 2017; Fuchs and Lauer, 2019; Romero and Trussell, 2022). The efferent synaptic inhibition at cochlear hair cells has been shown to involve α9α10-containing nicotinic receptors (Elgoyhen et al., 1994, 2001; Taranda et al., 2009). The activation of α9α10 nAChRs leads to increased Ca2+ influx, which subsequently activates Ca2+ dependent small conductance potassium SK2 channel (Dulon et al., 1998; Glowatzki and Fuchs, 2000; Oliver et al., 2000). This, in turn, leads to hyperpolarization of the cell membrane and hence, gain reduction (Roux et al., 2011). The main outcome of activating ACh release of this circuit is the suppression of sound-evoked responses, as demonstrated in Figure 3. This suppression leads to an elevated baseline cochlear threshold, and opposes acoustic injury caused by intense noise. In addition to this nicotinic effect, all five subtypes of muscarinic receptors are also found in the cochlea (Khan et al., 2002; Maison et al., 2010). Double deletion of M2/M4 mAChRs was shown to attenuate auditory responses of IHCs, which the authors suggest could decrease their vulnerability to acoustic injury (Maison et al., 2010). The muscarinic component is also suggested to act on cellular calcium-involving cascades to regulate the electromotility of OHC (Kalinec et al., 2000), presumably through shortening OHC and increasing motility amplitude (Kakehata et al., 1993; Dallos et al., 1997).

The effect of the olivo-cochlear reflex on the input–output encoding function of auditory fibers is well-known and has been thoroughly reviewed (Guinan, 1996; Künzel and Wagner, 2017). It has also been suggested that activation of the olivo-cochlear reflex could improve signal detection from noisy backgrounds by transiently enhancing acoustic gain in auditory fibers (Winslow and Sachs, 1988; Kawase et al., 1993). In terms of gain enhancement, one obvious benefit of modulation is to amplify responses to low input level sounds. The amplification of specific sound responses may have a particularly important function when acoustic information requires elevated neural sensitivity to ensure signal encoding. For example, in stimulus conditions where adapting neural responses to sound fall below threshold, temporary postsynaptic gain modulation may offset adaptive processes and preserve responses to reduced input amplitudes. We will highlight a few of the numerous examples type of modulation beyond the auditory periphery where cholinergic release has been documented to enhance or suppress responses at low stimulus intensities (Habbicht and Vater, 1996; Zhang et al., 2021).

### Acetylcholine in central nuclei: the cochlear nucleus

The coexistence of muscarinic and nicotinic AChRs in the CN was first described in 1966 (Comis and Whitfield, 1968). They showed that the administration of either atropine, a muscarinic antagonist or dihydro-β-erythroidine (DHβE), a nicotinic antagonist reversed the ACh-induced threshold lowering in cat CN. Anatomical evidence further supported the cholinergic innervation of the CN in cats (Osen and Roth, 1969; Godfrey et al., 1977). Rodent anatomical studies have documented the presence of ACh-related markers in CN of rats (Godfrey and Matschinsky, 1981; Godfrey et al., 1987; Sherriff and Henderson, 1994; Yao and Godfrey, 1995, 1996; Happe and Morley, 1998), chinchilla (Rasmussen, 1960, 1965), mice (Martin, 1981), guinea pigs (Mellott et al., 2011; Schofield et al., 2011) and gerbils (Gillet et al., 2018). Specifically, both mAChRs and nAChRs have been identified in the CN of rats (Morley et al., 1977; Hunt and Schmidt, 1978; Yao and Godfrey, 1995, 1996; Happe and Morley, 1998) and gerbils (Gillet et al., 2018).

**Figure 4:**
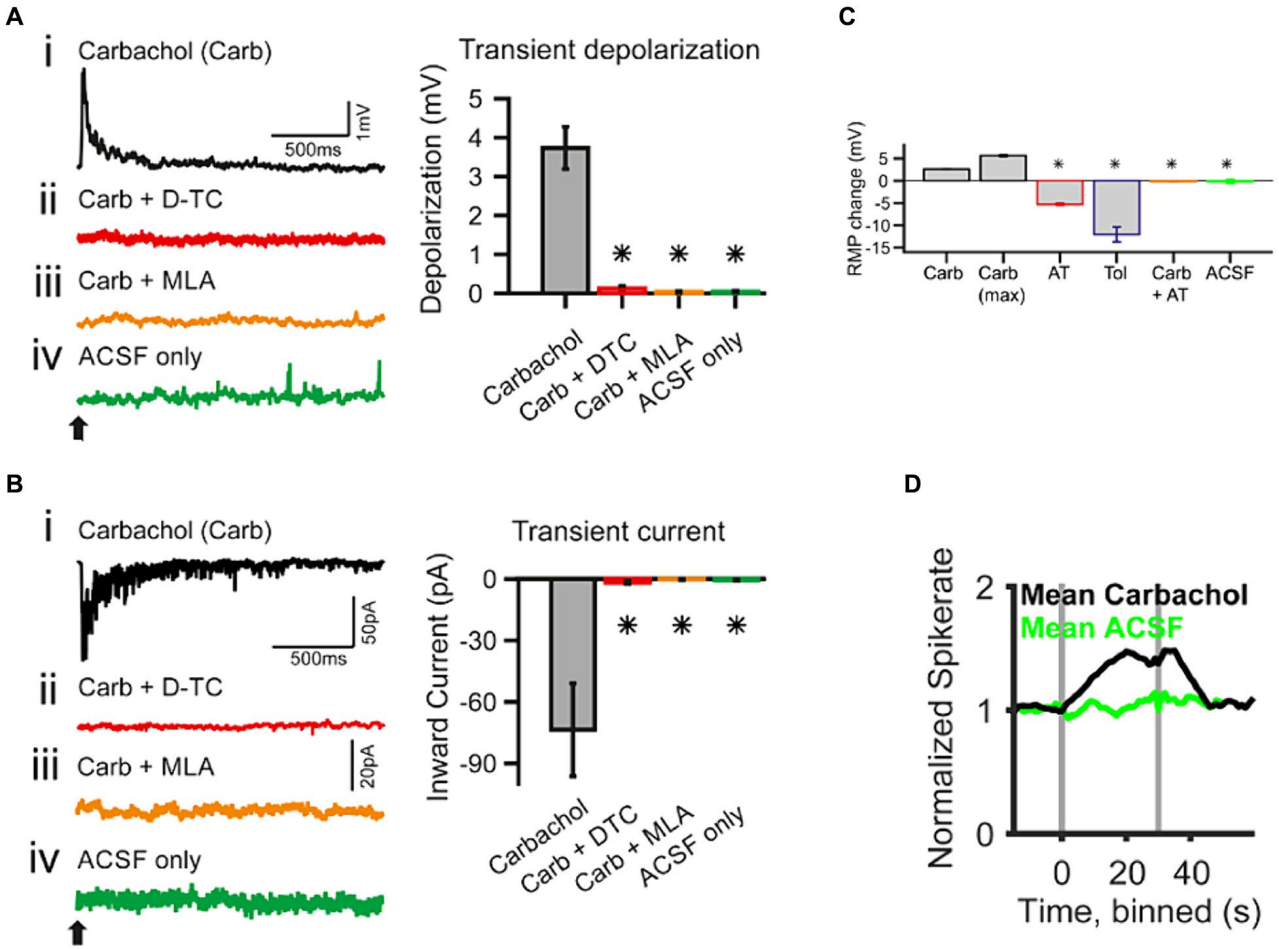
Whole-cell patch-clamp recording in spherical bushy cells (SBC) of the cochlear nucleus shows acetylcholine raises spiking probability [adapted from Goyer et al. (2016), with permission from eNeuro under BB-CY license]. **(A)** Transient effects of carbachol-mediated nAChR activation. The traces are current-clamp recordings with puff application of 500 μΜ carbachol (Carb; application time is marked by a *black arrow*). **(Ai–Aiv)** The transient depolarization elicited by the carbachol puff **(i)** was abolished when the slice had been superfused with 50 μΜ d-tubocurarine (D-TC), a general nAChR blocker **(ii)**; or with 20 nΜ methyllycaconitine (MLA), a specific α7 nAChR blocker **(iii)**; and with the puffing only the vehicle (ACSF) yielded no effect **(iv)**. **(B)** The traces are voltage-clamp recordings at −60 mV holding potential with carbachol application (application time marked by a black arrow). **(Bi–Biv)** SBCs showed a transient inward current upon carbachol application **(i)**, which was abolished in the presence of nicotinic blockers, D-TC **(ii)** and MLA **(iii)**; further, no current was observed upon puff application of the vehicle only **(iv)**. The population data for both type of recordings is shown on the right of each individual example. **(C)** Acetylcholine sets the SBC resting membrane potential (RMP) through muscarinic receptors. AT, atropine, muscarinic antagonist, 20 μΜ; Tol, tolterodine, muscarinic antagonist, 100 nΜ. **(D)** *In vivo* single unit recordings show that the spontaneous spike probability of SBC is increased by activating cholinergic signaling through iontophoresis of 5–500 mM carbachol (gray bars indicate the onset and offset of carbachol application). *p < 0.05.

Functionally, ACh has been shown to enhance acoustic gain in the CN. *In vivo* studies in the VCN demonstrated that ACh enhanced the tone-evoked response of VCN neurons (Caspary et al., 1983; Goyer et al., 2016). This effect steepened the slope in dynamic range of the RLF and as a result, the difference in firing rates evoked by adjacent intensities was magnified. This suggested an elevated sensitivity and enhanced neural discriminability between similar intensities. Specifically, in the spherical bushy cells (SBCs) of gerbil VCN, this effect is likely mediated by the activation of fast inward current associated with α7 subunit containing nAChRs (Figure 4; Goyer et al., 2016). Indeed, studies have shown that signal detection in noisy backgrounds is also improved in T stellate cells of VCN, through a transiently enhanced sensitivity to tones via a cholinergic input putatively evoked during the olivocochlear reflex (Fujino and Oertel, 2001).

Additional studies in other divisions of the CN have similarly shown that cholinergic activation suppressed sound-evoked responses. This was observed in DCN (Comis and Whitfield, 1968; Caspary et al., 1983); likely through M2 and/or M4 mAChRs (Chen et al., 1995). This suppression effect has been suggested to be involved in noise protection at the level of CN. As the synaptic targets of auditory nerve fibers, the diverse populations of CN neurons give rise to several parallel processing streams that ascend the auditory system. Among these types it has been suggested that the hyperactivity of fusiform cells indicates tinnitus. Intense sound-induced hyperactivity has been shown to be suppressed by carbachol in fusiform cells of DCN (Kaltenbach and Zhang, 2007; Manzoor et al., 2013). Additionally, intense tone exposure has been shown to upregulate the choline acetyltransferase activity in the hamster CN (Jin et al., 2006), suggesting another potentially protective mechanism mediated by intrinsic cholinergic circuitry.

### Acetylcholine in central nuclei: the superior olive

Several studies have identified markers of cholinergic signaling in the Superior Olive across a number of mammalian species. For example, mAChRs have been found in cat medial superior olive (MSO) through conventional autoradiographic receptor-binding of quinuclidinyl benzilate (QNB) (Glendenning and Baker, 1988). M3 muscarinic receptors were also found in LSO of both rat and guinea pig (Safieddine et al., 1996). In 2004, Happe and Morley demonstrated α7 nAChRs expression in rat SOC from radioactive mRNA *in situ* hybridization and from α-bungarotoxin binding (Happe and Morley, 2004). Similarly to that shown in CN, intense tones increased ChAT activity in the SOC (Godfrey et al., 2013), raising the possibility that the cholinergic network broadly protects against hearing damage throughout the system.

**Figure 5:**
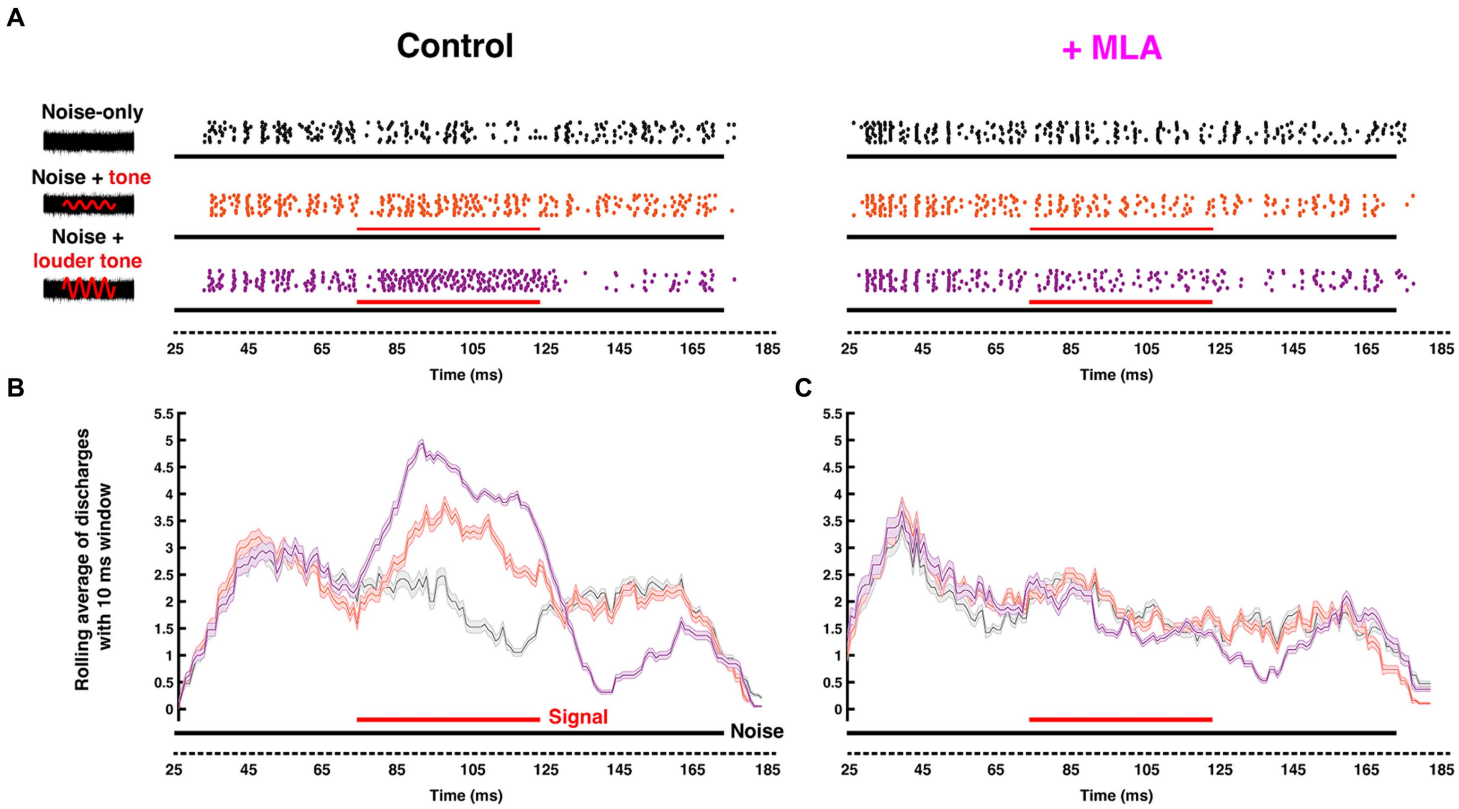
Endogenous cholinergic activity enhances signal detection of computational neurons in the gerbil auditory brainstem [Reproduced from Zhang et al. (2021), with permission from the Journal of Neuroscience under CC-BY license]. **(A)** Left: Schematic representation of the stimuli delivered to the ear canal of anesthetized gerbil. The stimuli include a 50 ms *CF* pure tone signal (*red*) of variable intensity embedded within a simultaneous 150-ms-wide-band frozen noise (*black*) presented at a fixed intensity. Amplitudes of the red sinusoidal curves schematically represent the variable selected tone intensities. Right: The corresponding raster plots with increasing signal/noise ratio, with each dot represent an action potential recorded from MNTB using *in vivo* extracellular recording with piggy-back multi-barrel electrodes, loaded with 20 mM methyllycaconitine (MLA), an α7-nAChR-specific antagonist. **(B,C)** Moving average (with a 10 ms shifting binning window) of action potentials fired before **(B)** and after **(C)** MLA administration. Solid black and red bars along the time axis represent the durations of noise and pure tone stimuli, respectively, where thickness indicates intensity. The neuron’s ability to differentiate pure-tone signal from wide-band noise was abolished after blocking α7 nAChRs.

Since many principal SOC neurons are functionally well-understood, the region provides fertile ground to empirically evaluate the contribution of cholinergic mechanisms to fundamental neural computations. Remarkably, physiological investigations of cholinergic modulation in the SOC were lacking until Huang and Trussell (2011) showed that blocking Kv7.5 elevated resting membrane potential of the presynaptic terminals in the Calyx of Held, regulating transmitter release. This finding suggested the involvement of muscarinic signaling due to the well-known modulatory coupling of mAChRs to Kv7 channel gating. Recently, we identified a postsynaptic mAChR mechanism in gerbil MNTB neurons that is limited to a developmental period up to and surrounding hearing onset (~ postnatal day 12) (Weimann et al., 2024). We showed that the postsynaptic activation mAChRs enhances MNTB excitability through suppression of a Kv7 conductance. This effect declines over the first week following hearing onset but appears to be partially offset by an emerging nicotinic response by around P18. In a separate *in vivo* study, we showed that by adulthood, nicotinic AChR activation fine-tunes sound intensity encoding performance of MNTB in a stimulus dependent manner. This effect is mediated by activation of both α7 and α4β2 nAChRs subtypes (Zhang et al., 2021). Further, Zhang et al. (2021) showed that the nAChR-induced increase in tonic firing improved MNTB neurons’ sensitivity to tones and discriminability between similar intensities. By using *in vivo* extracellular recording with pharmacological manipulations of nAChRs, this study also showed that activation of α7 or α4β2 nAChRs preferentially enhanced pure-tone evoked responses, relative to noise-driven responses (Figure 5). As a result, the ability of MNTB to detect pure-tones embedded in broadband noise was improved by cholinergic activation.

### Acetylcholine in central nuclei: the inferior colliculus

**Figure 6:**
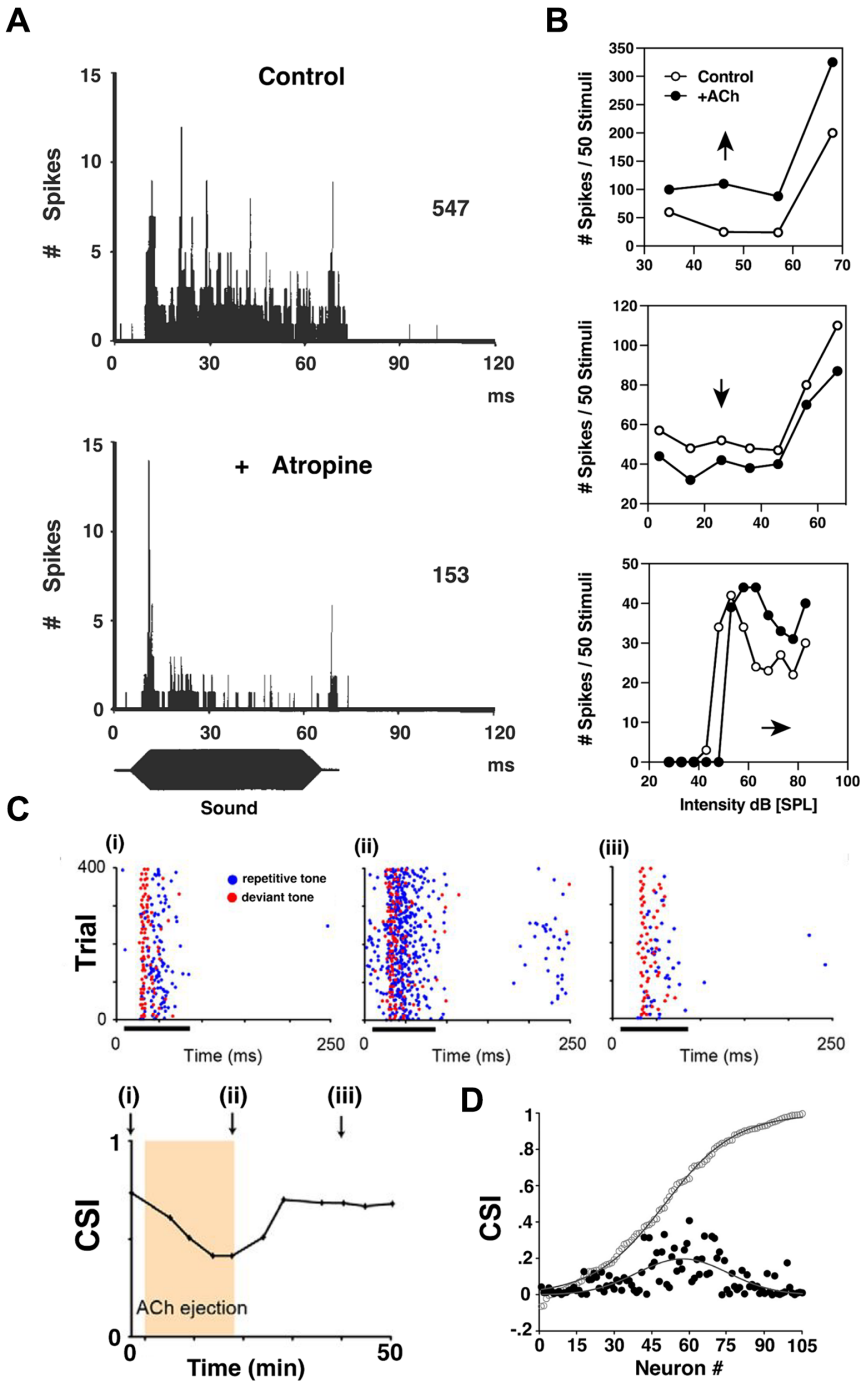
ACh influences input–output functions and stimulus-specific adaptation in the inferior colliculus. **(A)** Post-stimulus time histogram (PSTH) of discharges recorded from a bat IC neuron before (upper) and after (lower) atropine administration using multi-barrel electrodes. Blocking mAChRs of IC significantly decreased the tonic firing of the IC neurons during sound presentation. **(B)** The heterogeneity of cholinergic effects on bat IC neurons, reflected by the rate-level functions (RLFs) from three single units before and after ACh application. Upper: an upward shift of RLF indicates increased output (Spike count) without changing threshold; Middle: a downward shift of RLF indicates decrease output without affecting threshold; Lower: a parallel shift before saturation phase indicates an elevated threshold, and lowered maximum and saturation levels indicate decreased firing capabilities. [**A,B** are adapted and reproduced from Habbicht and Vater (1996); with permission from Elsevier; license # 5762610950216]. **(C)** Dot rasters of one rat IC neuron demonstrating stimulus-specific adaptation (SSA) under baseline **(i)**, ACh **(ii)**, and recovery **(iii)** conditions [adapted from Ayala and Malmierca (2015), with permission from the Journal of Neuroscience under BB-CY license]. During the experiment, the subject was presented with a standard, repetitive sound (blue) and a rare, deviant tone (red). The SSA was observable as a decrease in response to the standard *f1* tone *blue markers, left panel*, but not to a rare *f2* test tone *red markers*. The degree of SSA was quantified by the common SSA index (CSI, lower panel). The arrows in lower panel indicate the time point at which rasters are collected in panel **(i)–(iii)**. This particular neuron exhibits moderate levels of SSA, and the level of SSA was profoundly affected by ACh ejection due to elevated firing rate *middle panel*. On a population level shown in **(D)**, the strength of the effect of ACh depended on the baseline CSI. The baseline cumulative CSI values (open circles) among all IC neurons and the absolute response difference observed during ACh application (black circles, expressed in positive values) suggest that the SSA in IC neurons is broadly modulated by ACh, but is particularly strong for neurons in the moderate range of SSA magnitudes.

Similarly to lower auditory regions, ACh in the midbrain Inferior Colliculus (IC) produces excitatory effects on sound-evoked responses through both nicotinic and muscarinic receptors (Watanabe and Simada, 1973). Cholinergic modulation in IC was initially studied in the cat by monitoring *in vivo* extracellular spontaneous neural responses in IC (Curtis and Koizumi, 1961). Histologically, radioactive receptor-binding techniques have been used to confirm expression of metabotropic mAChRs in the IC of rat (Rotter et al., 1979), and cat (Glendenning and Baker, 1988), and ionotropic nAChRs in rat (Clarke et al., 1985; Morley and Happe, 2000). Furthermore, Sottile et al. (2017) showed that the β2-containing nAChRs are expressed in the rat GABAergic IC neurons, while the α4β2 nAChRs are expressed in non-GABAergic IC neurons. This finding was later substantiated by the Schofield group in a study showing that the cholinergic cells in the PMT contact both GABAergic and glutamatergic IC neurons (Noftz et al., 2020). Notably, a physiological study of α3β4 nAChRs in the auditory system characterized the influence of these receptors in the mouse IC (Rivera-Perez et al., 2021). Interestingly, Rivera-Perez et al. showed that activation of α3β4 nAChRs prolonged inward current and therefore extended the depolarization period for excitation, in contrast to the generally observed phenomenon that nAChR activation promotes short-duration depolarizations of cell membrane. Furthermore, extracellular recordings in the bat IC showed that cholinergic signaling affects the RLFs of IC neurons in a heterogenous fashion exhibiting either increased or in some cases, decreased, gain (Habbicht and Vater, 1996). In some neurons, the excitatory, upward shift of RLF was observed, with an elevated firing rate for both baseline intensity responses and over the dynamic range by the same magnitude. In other cases, the complementary effect was seen with a suppressive, downward shift of the RLF. There were also several cases where ACh elevated the auditory threshold of IC neurons and therefore, resulted in a parallel shift of the RLF (Figure 6). Together this constellation of varied effects appeared to extend modulatory capacity to influence encoding sensitivity to acoustic input.

One key finding demonstrated a more targeted and specific modulation of response gain in the IC. An innovative and foundational study by Ayala and Malmierca (2015) showed that ACh also contributes to stimulus-specific adaptation (SSA) in rat IC. They repetitively presented two separate tones while monitoring single-neuron activity *in vivo*. One tone (*f1*) was repeated at a high repetition rate, while the *‘test tone’(f2)* was presented only rarely. During 75 ms bouts of sound presentation, *f1* was presented with 90% probability of occurrence while *f2* was presented rarely at just 10% probability. Both tones were presented near the neuron’s characteristic frequency (*CF*), and each evoked similar firing patterns. SSA typically manifested as a decreased response to the repetitive tone (*f1*) while the test tone (*f2*) response was unaffected (Figure 6C). The SSA magnitude was assessed by comparing the rare-tone evoked responses to those of the repetitive tones over the course of each trial. Interestingly, for neurons showing moderate SSA to the repetitive tone (*f1*), ACh preferentially enhanced these adapted responses relative to test tone responses. Ayala and Malmierca (2015) then showed that this differential influence was generally attributable to mAChR activation. Significantly, this study demonstrated that ACh has the capacity to enhance auditory encoding in a highly specific manner by differentially modulating single neuron responses to very similar stimuli.

Another important finding of cholinergic effects in IC revealed its role in plasticity of stimulus feature selectivity. Ji and Suga (2009) characterized a shift of best frequency (BF) tuning in IC neurons of bats induced by acoustic conditioning. Their study showed that applying mAChRs antagonist abolished this BF shift (Ji and Suga, 2009), but activating mAChRs significantly *augmented* the BF shift (Ji et al., 2001). Gao and Suga (2000) suggested that the corticofugal efferent feedback to IC, exhibits cholinergic plasticity that, in turn, contributes to the magnitude of cortical neuron BF plasticity. This finding implicates cholinergic circuitry in associative learning. Taken together, both Ayala and Malmierca (2015) as well as the Suga laboratory studies demonstrated that ACh can mediate very subtle stimulus-specific effects that extend its modulatory potential well beyond simple gain control mechanisms.

## Conclusion

**Figure 7:**
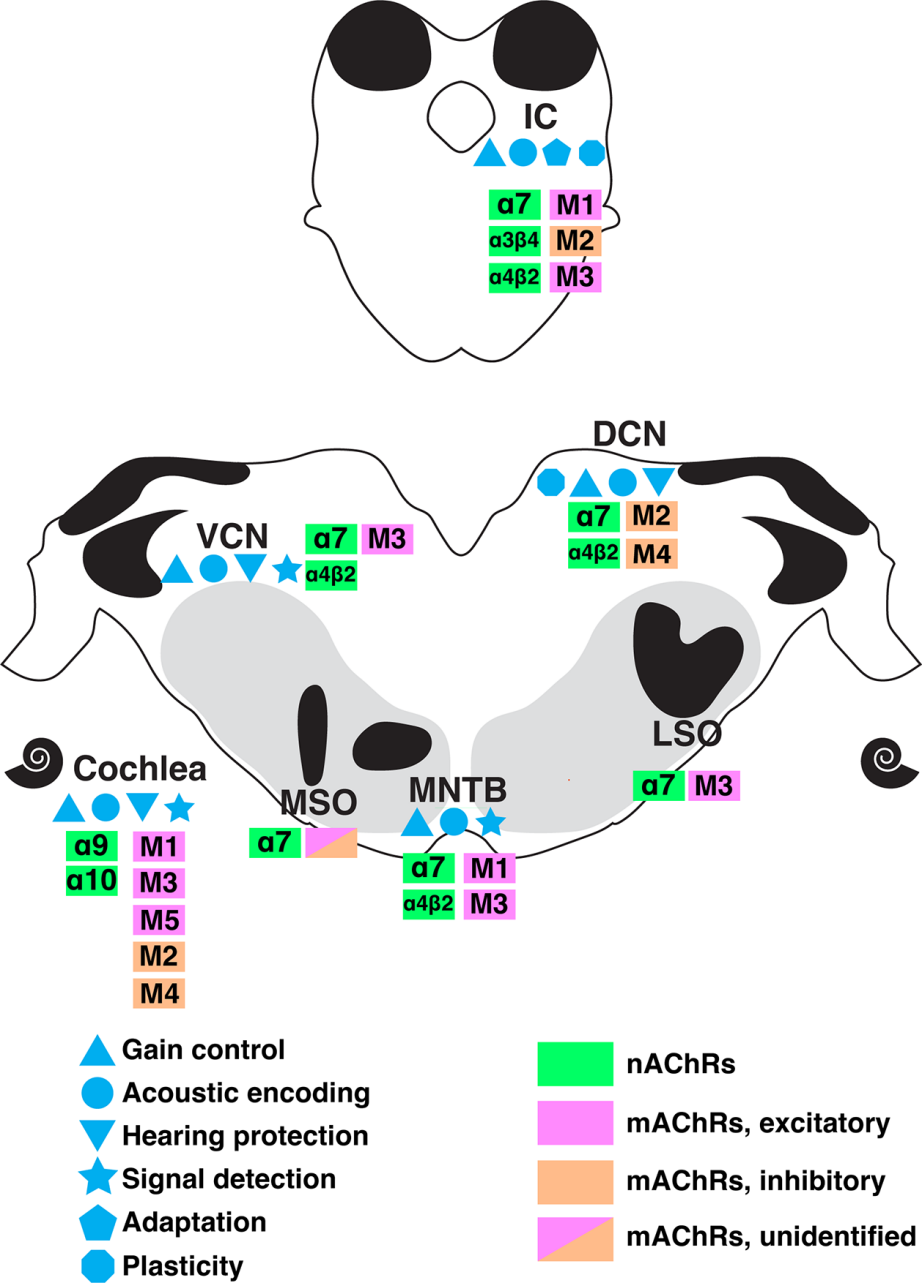
A summary of the currently understood distribution of cholinergic receptor subtypes, expression patterns, and functional roles along the ascending auditory pathway as indicated by colors and symbols shown. DCN, dorsal cochlear nucleus; IC, inferior colliculus; LSO, lateral superior olive; MNTB, medial nucleus of trapezoid body; MSO, medial superior olive; VCN, ventral cochlear nucleus.

Cholinergic circuitry has long been implicated in higher order functions such as attention and sensory gating, particularly in forebrain structures. However, recent emerging work has convincingly showed that ACh is also intricately involved in serving even the most basic functions of sensory encoding throughout the auditory pathway including lower order processing regions. In this review, we summarized a wide range of physiologically important cholinergic impacts on neural computation in the brainstem auditory system. These functions include acoustic gain control, sound encoding, noise protection, signal-in-noise detection and intensity adaptation. Figure 7 summarizes the known receptor expression patterns and functional roles for cholinergic modulation in the lower auditory system as evidenced in the literature to date. M1-M5 mAChRs and α7, α4β2, α9α10, α3β4-containing nAChRs have been demonstrated to mediate these wide ranging and impactful computational functions. The variety of acetylcholine receptors expressed in these nuclei further suggests the prevalence and significance of neuromodulation at early stage of sound processing. Overall, these findings underscore a general view that sound processing depends on a sophisticated coordination of synaptic inputs from overlapping afferent, efferent, and modulatory circuitry. It is clear from the emerging work highlighted here, that there remains much more to be discovered regarding modulatory influences on auditory computations than is currently understood.

## Author contributions

CZ: Writing – original draft, Writing – review & editing. RB: Writing – original draft, Writing – review & editing.

## References

[ref1] Adams J. C. (1979). Ascending projections to the inferior colliculus. J. Comp. Neurol. 183, 519–538. doi: 10.1002/cne.901830305, PMID: 759446

[ref2] Akers A. T. Cooper S. Y. Baumgard Z. J. Casinelli G. P. Avelar A. J. Henderson B. J. (2020). Upregulation of nAChRs and changes in excitability on VTA dopamine and GABA neurons correlates to changes in nicotine-reward-related behavior. 7, ENEURO.0189–ENEU20.2020. doi: 10.1523/ENEURO.0189-20.2020 PMC7568605 32988984

[ref3] Andrezik J. A. Chan-Palay V. Palay S. L. (1981). The nucleus paragigantocellularis lateralis in the rat. Demonstration of afferents by the retrograde transport of horseradish peroxidase. Anat Embryol (Berl) 161, 373–390. doi: 10.1007/BF00316049 7247035

[ref4] Arnesen A. R. Osen K. K. (1978). The cochlear nerve in the cat: topography, cochleotopy, and fiber spectrum. J. Comp. Neurol. 178, 661–678. doi: 10.1002/cne.901780405, PMID: 632375

[ref5] Ashe J. H. McKenna T. M. Weinberger N. M. (1989). Cholinergic modulation of frequency receptive fields in auditory cortex: II. Frequency-specific effects of anticholinesterases provide evidence for a modulatory action of endogenous ACh. Synapse 4, 44–54. doi: 10.1002/syn.890040106, PMID: 2772838

[ref9001] Ayala Y. A. Malmierca M. S. (2015). Cholinergic modulation of stimulus-specific adaptation in the inferior colliculus. J. Neurosci. 35, 12261–12272. 26338336 10.1523/JNEUROSCI.0909-15.2015 PMC6605313

[ref6] Baeyer A. (1867). I. Ueber das Neurin. Justus Liebigs Ann. Chem. 142, 322–326. doi: 10.1002/jlac.18671420311

[ref7] Beebe N. L. Herrera Y. N. Noftz W. A. Roberts M. T. Schofield B. R. (2023). Characterization of three cholinergic inputs to the cochlear nucleus. J. Chem. Neuroanat. 131:102284. doi: 10.1016/j.jchemneu.2023.102284, PMID: 37164181 PMC10330717

[ref8] Beebe N. L. Zhang C. Burger R. M. Schofield B. R. (2021). Multiple sources of cholinergic input to the superior Olivary complex. Front. Neural. Circuits 15:715369. doi: 10.3389/fncir.2021.715369, PMID: 34335196 PMC8319744

[ref9] Beker F. Weber M. Fink R. H. Adams D. J. (2003). Muscarinic and nicotinic ACh receptor activation differentially mobilize Ca2+ in rat intracardiac ganglion neurons. J. Neurophysiol. 90, 1956–1964. doi: 10.1152/jn.01079.2002, PMID: 12761283

[ref10] Bizley J. K. (2017). “Chapter 26 - audition” in Conn's Translational Neuroscience. ed. Conn P. M. (San Diego: Academic Press), 579–598.

[ref11] Bosch D. Schmid S. (2006). Activation of muscarinic cholinergic receptors inhibits giant neurones in the caudal pontine reticular nucleus. Eur. J. Neurosci. 24, 1967–1975. doi: 10.1111/j.1460-9568.2006.05085.x, PMID: 17040474

[ref12] Brown D. A. (2018). Regulation of neural ion channels by muscarinic receptors. Neuropharmacology 136, 383–400. doi: 10.1016/j.neuropharm.2017.11.024, PMID: 29154951

[ref13] Brown D. A. Adams P. R. (1980). Muscarinic suppression of a novel voltage-sensitive K+ current in a vertebrate neurone. Nature 283, 673–676. doi: 10.1038/283673a0, PMID: 6965523

[ref14] Bruce Warr W. (1995). “Parallel ascending pathways from the Cochlear nucleus: neuroanatomical evidence of functional specialization” in Contributions to sensory physiology. ed. Neff W. D. (Elsevier), 1–38.

[ref15] Brugge J. F. Howard M. A. (2002). “Hearing” in Encyclopedia of the human brain. ed. Ramachandran V. S. (New York: Academic Press), 429–448.

[ref16] Burger R. M. Rubel E. (2008). The senses: a comprehensive reference. Ed. D. Oertel. 613–630.

[ref17] Caminos E. Garcia-Pino E. Martinez-Galan J. R. Juiz J. M. (2007). The potassium channel KCNQ5/Kv7.5 is localized in synaptic endings of auditory brainstem nuclei of the rat. J. Comp. Neurol. 505, 363–378. doi: 10.1002/cne.21497, PMID: 17912742

[ref18] Caspary D. M. Havey D. C. Faingold C. L. (1983). Effects of acetylcholine on cochlear nucleus neurons. Exp. Neurol. 82, 491–498. doi: 10.1016/0014-4886(83)90419-3 6628633

[ref19] Caulfield M. P. Birdsall N. J. (1998). International Union of Pharmacology. XVII. Classification of muscarinic acetylcholine receptors. Pharmacol. Rev. 50, 279–290, PMID: 9647869

[ref20] Changeux J. P. Paas Y. (2009). “Nicotinic acetylcholine receptors” in Encyclopedia of neuroscience. ed. Squire L. R. (Oxford: Academic Press), 1129–1133.

[ref21] Chen K. Waller H. J. Godfrey D. A. (1995). Muscarinic receptor subtypes in rat dorsal cochlear nucleus. Hear. Res. 89, 137–145. doi: 10.1016/0378-5955(95)00131-6, PMID: 8600118

[ref22] Ciuman R. R. (2010). The efferent system or olivocochlear function bundle - fine regulator and protector of hearing perception. Int. J. Biomed. Sci. 6, 276–288. doi: 10.59566/IJBS.2010.6276 23675203 PMC3615293

[ref23] Clarke P. B. Schwartz R. D. Paul S. M. Pert C. B. Pert A. (1985). Nicotinic binding in rat brain: autoradiographic comparison of [3H]acetylcholine, [3H]nicotine, and [125I]-alpha-bungarotoxin. J. Neurosci. 5, 1307–1315. doi: 10.1523/JNEUROSCI.05-05-01307.1985, PMID: 3998824 PMC6565049

[ref24] Coggan J. S. Purnyn S. L. Knoper S. R. Kreulen D. L. (1994). Muscarinic inhibition of two potassium currents in guinea-pig prevertebral neurons: differentiation by extracellular cesium. Neuroscience 59, 349–361. doi: 10.1016/0306-4522(94)90601-7, PMID: 8008197

[ref25] Comis S. D. Whitfield I. C. (1968). Influence of centrifugal pathways on unit activity in the cochlear nucleus. J. Neurophysiol. 31, 62–68. doi: 10.1152/jn.1968.31.1.62, PMID: 5640740

[ref26] Curtis D. R. Koizumi K. (1961). Chemical transmitter substances in brain stem of cat. J. Neurophysiol. 24, 80–90. doi: 10.1152/jn.1961.24.1.80, PMID: 13718944

[ref27] Dale H. H. (1914). The action of certain esters and ethers of choline, and their relation to MUSCARINE. J. Pharmacol. Exp. Ther. 6, 147–190.

[ref28] Dallos P. He D. Z. Lin X. Sziklai I. Mehta S. Evans B. N. (1997). Acetylcholine, outer hair cell electromotility, and the cochlear amplifier. J. Neurosci. 17, 2212–2226. doi: 10.1523/JNEUROSCI.17-06-02212.1997, PMID: 9045745 PMC6793750

[ref29] Darrow K. N. Maison S. F. Liberman M. C. (2006). Cochlear efferent feedback balances interaural sensitivity. Nat. Neurosci. 9, 1474–1476. doi: 10.1038/nn1807, PMID: 17115038 PMC1806686

[ref30] Darrow K. N. Maison S. F. Liberman M. C. (2007). Selective removal of lateral olivocochlear efferents increases vulnerability to acute acoustic injury. J. Neurophysiol. 97, 1775–1785. doi: 10.1152/jn.00955.2006 17093118 PMC1805782

[ref31] Davis K. A. (2005). Contralateral effects and binaural interactions in dorsal cochlear nucleus. J. Assoc. Res. Otolaryngol. 6, 280–296. doi: 10.1007/s10162-005-0008-5, PMID: 16075189 PMC2504593

[ref32] Dell'Acqua M. L. Carroll R. C. Peralta E. G. (1993). Transfected m2 muscarinic acetylcholine receptors couple to G alpha i2 and G alpha i3 in Chinese hamster ovary cells. Activation and desensitization of the phospholipase C signaling pathway. J. Biol. Chem. 268, 5676–5685. doi: 10.1016/S0021-9258(18)53372-X, PMID: 8449930

[ref33] Dulon D. Luo L. Zhang C. Ryan A. F. (1998). Expression of small-conductance calcium-activated potassium channels (SK) in outer hair cells of the rat cochlea. Eur. J. Neurosci. 10, 907–915. doi: 10.1046/j.1460-9568.1998.00098.x, PMID: 9753158

[ref34] Eglen R. M. (2006). Muscarinic receptor subtypes in neuronal and non-neuronal cholinergic function. Auton. Autacoid Pharmacol. 26, 219–233. doi: 10.1111/j.1474-8673.2006.00368.x 16879488

[ref35] Elgoyhen A. B. Johnson D. S. Boulter J. Vetter D. E. Heinemann S. (1994). Alpha 9: an acetylcholine receptor with novel pharmacological properties expressed in rat cochlear hair cells. Cell 79, 705–715. doi: 10.1016/0092-8674(94)90555-X, PMID: 7954834

[ref36] Elgoyhen A. B. Vetter D. E. Katz E. Rothlin C. V. Heinemann S. F. Boulter J. (2001). alpha10: a determinant of nicotinic cholinergic receptor function in mammalian vestibular and cochlear mechanosensory hair cells. Proc. Natl. Acad. Sci. U. S. A. 98, 3501–3506. doi: 10.1073/pnas.051622798, PMID: 11248107 PMC30682

[ref37] Froemke R. C. Merzenich M. M. Schreiner C. E. (2007). A synaptic memory trace for cortical receptive field plasticity. Nature 450, 425–429. doi: 10.1038/nature06289, PMID: 18004384

[ref38] Fuchs P. A. Lauer A. M. (2019). Efferent inhibition of the cochlea. Cold Spring Harb. Perspect. Med. 9:a033530. doi: 10.1101/cshperspect.a033530, PMID: 30082454 PMC6496333

[ref39] Fujino K. Oertel D. (2001). Cholinergic modulation of stellate cells in the mammalian ventral cochlear nucleus. J. Neurosci. 21, 7372–7383. doi: 10.1523/JNEUROSCI.21-18-07372.2001, PMID: 11549747 PMC6763002

[ref9002] Gao E. Suga N. (2000). Experience-dependent plasticity in the auditory cortex and the inferior colliculus of bats: role of the corticofugal system. Proc. Natl. Acad. Sci. U. S. A. 97, 8081–8086. 10884432 10.1073/pnas.97.14.8081 PMC16673

[ref40] Garcia-Pino E. Caminos E. Juiz J. M. (2010). KCNQ5 reaches synaptic endings in the auditory brainstem at hearing onset and targeting maintenance is activity-dependent. J. Comp. Neurol. 518, 1301–1314. doi: 10.1002/cne.22276, PMID: 20151361

[ref41] Gillet C. Goyer D. Kurth S. Griebel H. Kuenzel T. (2018). Cholinergic innervation of principal neurons in the cochlear nucleus of the Mongolian gerbil. J. Comp. Neurol. 526, 1647–1661. doi: 10.1002/cne.24433, PMID: 29574885

[ref42] Glendenning K. K. Baker B. N. (1988). Neuroanatomical distribution of receptors for three potential inhibitory neurotransmitters in the brainstem auditory nuclei of the cat. J. Comp. Neurol. 275, 288–308. doi: 10.1002/cne.902750210, PMID: 2851616

[ref43] Glendenning K. K. Brunso-Bechtold J. K. Thompson G. C. Masterton R. B. (1981). Ascending auditory afferents to the nuclei of the lateral lemniscus. J. Comp. Neurol. 197, 673–703. doi: 10.1002/cne.901970409, PMID: 7229133

[ref44] Glendenning K. K. Hutson K. A. Nudo R. J. Masterton R. B. (1985). Acoustic chiasm II: anatomical basis of binaurality in lateral superior olive of cat. J. Comp. Neurol. 232, 261–285. doi: 10.1002/cne.902320210, PMID: 3973093

[ref45] Glendenning K. K. Masterton R. B. Baker B. N. Wenthold R. J. (1991). Acoustic chiasm. III: nature, distribution, and sources of afferents to the lateral superior olive in the cat. J. Comp. Neurol. 310, 377–400. doi: 10.1002/cne.903100308, PMID: 1723989

[ref46] Glowatzki E. Fuchs P. A. (2000). Cholinergic synaptic inhibition of inner hair cells in the neonatal mammalian cochlea. Science 288, 2366–2368. doi: 10.1126/science.288.5475.2366, PMID: 10875922

[ref47] Godfrey D. A. Kaltenbach J. A. Chen K. Ilyas O. (2013). Choline acetyltransferase activity in the hamster central auditory system and long-term effects of intense tone exposure. J. Neurosci. Res. 91, 987–996. doi: 10.1002/jnr.23227, PMID: 23605746 PMC4469331

[ref48] Godfrey D. A. Matschinsky F. M. (1981). Quantitative distribution of choline acetyltransferase and acetylcholinesterase activities in the rat cochlear nucleus. J. Histochem. Cytochem. 29, 720–730. doi: 10.1177/29.6.7252132 7252132

[ref49] Godfrey D. A. Park-Hellendall J. L. Dunn J. D. Ross C. D. (1987). Effects of trapezoid body and superior olive lesions on choline acetyltransferase activity in the rat cochlear nucleus. Hear. Res. 28, 253–270. doi: 10.1016/0378-5955(87)90053-0, PMID: 3654393

[ref50] Godfrey D. A. Williams A. D. Matschinsky F. M. (1977). Quantitative histochemical mapping of enzymes of the cholinergic system in cat cochlear nucleus. J. Histochem. Cytochem. 25, 397–416. doi: 10.1177/25.6.69653, PMID: 69653

[ref51] Goldberg J. M. Brown P. B. (1969). Response of binaural neurons of dog superior olivary complex to dichotic tonal stimuli: some physiological mechanisms of sound localization. J. Neurophysiol. 32, 613–636. doi: 10.1152/jn.1969.32.4.613, PMID: 5810617

[ref52] Gomez-Nieto R. Rubio M. E. Lopez D. E. (2008). Cholinergic input from the ventral nucleus of the trapezoid body to cochlear root neurons in rats. J. Comp. Neurol. 506, 452–468. doi: 10.1002/cne.21554, PMID: 18041785

[ref53] Goyer D. Kurth S. Gillet C. Keine C. Rubsamen R. Kuenzel T. (2016). Slow cholinergic modulation of spike probability in ultra-fast time-coding sensory neurons. eNeuro 3, ENEURO.0186–ENEU16.2016. doi: 10.1523/ENEURO.0186-16.2016 27699207 PMC5035776

[ref54] Grothe B. Pecka M. (2014). The natural history of sound localization in mammals--a story of neuronal inhibition. Front. Neural Circuits 8:116. doi: 10.3389/fncir.2014.00116 25324726 PMC4181121

[ref55] Grothe B. Sanes D. H. (1993). Bilateral inhibition by glycinergic afferents in the medial superior olive. J. Neurophysiol. 69, 1192–1196. doi: 10.1152/jn.1993.69.4.1192, PMID: 8492158

[ref56] Guinan J. J. (1996). “Physiology of Olivocochlear Efferents” in The cochlea. eds. Dallos P. Popper A. N. Fay R. R. (New York, NY: Springer New York), 435–502.

[ref57] Guinan J. J. (2018). Olivocochlear efferents: their action, effects, measurement and uses, and the impact of the new conception of cochlear mechanical responses. Hear. Res. 362, 38–47. doi: 10.1016/j.heares.2017.12.012, PMID: 29291948 PMC5911200

[ref58] Habbicht H. Vater M. (1996). A microiontophoretic study of acetylcholine effects in the inferior colliculus of horseshoe bats: implications for a modulatory role. Brain Res. 724, 169–179. doi: 10.1016/0006-8993(96)00224-7, PMID: 8828565

[ref59] Hackney C. M. Osen K. K. Kolston J. (1990). Anatomy of the cochlear nuclear complex of guinea pig. Anat Embryol 182, 123–149. doi: 10.1007/BF00174013, PMID: 2244686

[ref60] Hallanger A. E. Levey A. I. Lee H. J. Rye D. B. Wainer B. H. (1987). The origins of cholinergic and other subcortical afferents to the thalamus in the rat. J. Comp. Neurol. 262, 105–124. doi: 10.1002/cne.902620109 2442206

[ref61] Hamada S. Houtani T. Trifonov S. Kase M. Maruyama M. Shimizu J.. (2010). Histological determination of the areas enriched in cholinergic terminals and M2 and M3 muscarinic receptors in the mouse central auditory system. Anat. Rec. (Hoboken) 293, 1393–1399. doi: 10.1002/ar.21186, PMID: 20665816

[ref62] Hammond C. (2015). “Chapter 8 - the ionotropic nicotinic acetylcholine receptors” in Cellular and molecular neurophysiology. ed. Hammond C.. 4th ed (Boston: Academic Press), 173–197.

[ref63] Happe H. K. Morley B. J. (1998). Nicotinic acetylcholine receptors in rat cochlear nucleus: [125I]-alpha-bungarotoxin receptor autoradiography and in situ hybridization of alpha 7 nAChR subunit mRNA. J. Comp. Neurol. 397, 163–180. doi: 10.1002/(SICI)1096-9861(19980727)397:2<163::AID-CNE2>3.0.CO;2-Z, PMID: 9658282

[ref64] Happe H. K. Morley B. J. (2004). Distribution and postnatal development of alpha 7 nicotinic acetylcholine receptors in the rodent lower auditory brainstem. Brain Res. Dev. Brain Res. 153, 29–37. doi: 10.1016/j.devbrainres.2004.07.004, PMID: 15464215

[ref65] Harrison J. M. Howe M. E. (1974). “Anatomy of the descending auditory system (mammalian)” in Auditory system: Anatomy physiology. eds. Ades H. W. Axelsson A. Baird I. L. V Békésy G. Boord R. L. CBG C.. (Berlin, Heidelberg: Springer Berlin Heidelberg), 363–388.

[ref66] Harrison J. M. Irving R. (1966). Ascending connections of the anterior ventral cochlear nucleus in the rat. J. Comp. Neurol. 126, 51–63. doi: 10.1002/cne.901260105, PMID: 5935369

[ref67] Heckers S. Geula C. Mesulam M. M. (1992). Cholinergic innervation of the human thalamus: dual origin and differential nuclear distribution. J. Comp. Neurol. 325, 68–82. doi: 10.1002/cne.903250107, PMID: 1282919

[ref68] Henkel C. K. (2018). “Chapter 21 - the auditory system” in Fundamental neuroscience for basic and clinical applications. eds. Haines D. E. Mihailoff G. A.. 5th ed (Elsevier), 306–319.e301.

[ref9003] Huang H. Trussell L. O. (2011). KCNQ5 channels control resting properties and release probability of a synapse. Nat. Neurosci. 14, 840–847. 21666672 10.1038/nn.2830 PMC3133966

[ref69] Hunt S. Schmidt J. (1978). Some observations on the binding patterns of alpha-bungarotoxin in the central nervous system of the rat. Brain Res. 157, 213–232. doi: 10.1016/0006-8993(78)90025-2 719523

[ref70] Irie T. Fukui I. Ohmori H. (2006). Activation of GIRK channels by muscarinic receptors and group II metabotropic glutamate receptors suppresses Golgi cell activity in the cochlear nucleus of mice. J. Neurophysiol. 96, 2633–2644. doi: 10.1152/jn.00396.2006, PMID: 16855110

[ref71] Ishii M. Kurachi Y. (2006). Muscarinic acetylcholine receptors. Curr. Pharm. Des. 12, 3573–3581. doi: 10.2174/138161206778522056 17073660

[ref72] Itier V. Bertrand D. (2001). Neuronal nicotinic receptors: from protein structure to function. FEBS Lett. 504, 118–125. doi: 10.1016/S0014-5793(01)02702-8 11532443

[ref73] Ito T. Bishop D. C. Oliver D. L. (2016). Functional organization of the local circuit in the inferior colliculus. Anat. Sci. Int. 91, 22–34. doi: 10.1007/s12565-015-0308-8, PMID: 26497006 PMC4846595

[ref74] James N. M. Gritton H. J. Kopell N. Sen K. Han X. (2019). Muscarinic receptors regulate auditory and prefrontal cortical communication during auditory processing. Neuropharmacology 144, 155–171. doi: 10.1016/j.neuropharm.2018.10.027, PMID: 30352212 PMC6400225

[ref75] Ji W. Gao E. Suga N. (2001). Effects of acetylcholine and atropine on plasticity of central auditory neurons caused by conditioning in bats. J. Neurophysiol. 86, 211–225. doi: 10.1152/jn.2001.86.1.211, PMID: 11431503

[ref76] Ji W. Suga N. (2009). Tone-specific and nonspecific plasticity of inferior colliculus elicited by pseudo-conditioning: role of acetylcholine and auditory and somatosensory cortices. J. Neurophysiol. 102, 941–952. doi: 10.1152/jn.00222.2009, PMID: 19474174 PMC2724339

[ref77] Jin Y. M. Godfrey D. A. Wang J. Kaltenbach J. A. (2006). Effects of intense tone exposure on choline acetyltransferase activity in the hamster cochlear nucleus. Hear. Res. 216-217, 168–175. doi: 10.1016/j.heares.2006.02.002, PMID: 16549284

[ref78] Kakehata S. Nakagawa T. Takasaka T. Akaike N. (1993). Cellular mechanism of acetylcholine-induced response in dissociated outer hair cells of guinea-pig cochlea. J. Physiol. 463, 227–244. doi: 10.1113/jphysiol.1993.sp019592, PMID: 7504105 PMC1175341

[ref79] Kalinec F. Zhang M. Urrutia R. Kalinec G. (2000). Rho GTPases mediate the regulation of cochlear outer hair cell motility by acetylcholine. J. Biol. Chem. 275, 28000–28005. doi: 10.1074/jbc.M004917200 10862776

[ref80] Kaltenbach J. A. Zhang J. (2007). Intense sound-induced plasticity in the dorsal cochlear nucleus of rats: evidence for cholinergic receptor upregulation. Hear. Res. 226, 232–243. doi: 10.1016/j.heares.2006.07.001, PMID: 16914276

[ref81] Kamiya H. Itoh K. Yasui Y. Ino T. Mizuno N. (1988). Somatosensory and auditory relay nucleus in the rostral part of the ventrolateral medulla: a morphological study in the cat. J. Comp. Neurol. 273, 421–435. doi: 10.1002/cne.902730311, PMID: 2463282

[ref82] Kawai H. Lazar R. Metherate R. (2007). Nicotinic control of axon excitability regulates thalamocortical transmission. Nat. Neurosci. 10, 1168–1175. doi: 10.1038/nn1956, PMID: 17704774

[ref83] Kawase T. Delgutte B. Liberman M. C. (1993). Antimasking effects of the olivocochlear reflex. II. Enhancement of auditory-nerve response to masked tones. J. Neurophysiol. 70, 2533–2549. doi: 10.1152/jn.1993.70.6.2533, PMID: 8120597

[ref84] Khan K. M. Drescher M. J. Hatfield J. S. Khan A. M. Drescher D. G. (2002). Muscarinic receptor subtypes are differentially distributed in the rat cochlea. Neuroscience 111, 291–302. doi: 10.1016/S0306-4522(02)00020-9, PMID: 11983315

[ref85] Kharkovets T. Hardelin J. P. Safieddine S. Schweizer M. El-Amraoui A. Petit C.. (2000). KCNQ4, a K+ channel mutated in a form of dominant deafness, is expressed in the inner ear and the central auditory pathway. Proc. Natl. Acad. Sci. USA 97, 4333–4338. doi: 10.1073/pnas.97.8.4333, PMID: 10760300 PMC18242

[ref86] Kiss A. Majorossy K. (1983). Neuron morphology and synaptic architecture in the medial superior olivary nucleus. Light- and electron microscope studies in the cat. Exp. Brain Res. 52, 315–327. doi: 10.1007/BF00238026, PMID: 6653694

[ref87] Koyama Y. Jodo E. Kayama Y. (1994). Sensory responsiveness of "broad-spike" neurons in the laterodorsal tegmental nucleus, locus coeruleus and dorsal raphe of awake rats: implications for cholinergic and monoaminergic neuron-specific responses. Neuroscience 63, 1021–1031. doi: 10.1016/0306-4522(94)90569-X, PMID: 7700507

[ref88] Kudo M. Niimi K. (1980). Ascending projections of the inferior colliculus in the cat: an autoradiographic study. J. Comp. Neurol. 191, 545–556. doi: 10.1002/cne.901910403 7419733

[ref89] Kunnath A. J. Gifford R. H. Wallace M. T. (2023). Cholinergic modulation of sensory perception and plasticity. Neurosci. Biobehav. Rev. 152:105323. doi: 10.1016/j.neubiorev.2023.105323 37467908 PMC10424559

[ref90] Künzel T. Wagner H. (2017). Cholinergic top-down influences on the auditory brainstem. e-Neuroforum 23, 35–44. doi: 10.1515/nf-2016-A107

[ref91] Leach N. D. Nodal F. R. Cordery P. M. King A. J. Bajo V. M. (2013). Cortical cholinergic input is required for normal auditory perception and experience-dependent plasticity in adult ferrets. J. Neurosci. 33, 6659–6671. doi: 10.1523/JNEUROSCI.5039-12.2013, PMID: 23575862 PMC3682393

[ref92] Liang K. Poytress B. S. Chen Y. Leslie F. M. Weinberger N. M. Metherate R. (2006). Neonatal nicotine exposure impairs nicotinic enhancement of central auditory processing and auditory learning in adult rats. Eur. J. Neurosci. 24, 857–866. doi: 10.1111/j.1460-9568.2006.04945.x 16848798

[ref93] Liang K. Poytress B. S. Weinberger N. M. Metherate R. (2008). Nicotinic modulation of tone-evoked responses in auditory cortex reflects the strength of prior auditory learning. Neurobiol. Learn. Mem. 90, 138–146. doi: 10.1016/j.nlm.2008.02.006 18378471 PMC2464281

[ref94] Loewi O. Navratil E. (1926). Über humorale Übertragbarkeit der Herznervenwirkung. Pflugers Arch. Gesamte Physiol. Menschen Tiere 214-214, 678–688. doi: 10.1007/BF01741946

[ref95] Maclaine K. D. Llano D. A. (2020). “2.45 - the aging central auditory system” in The senses: A comprehensive reference. ed. Fritzsch B.. 2nd ed (Oxford: Elsevier), 884–895.

[ref96] Madison D. V. Lancaster B. Nicoll R. A. (1987). Voltage clamp analysis of cholinergic action in the hippocampus. J. Neurosci. 7, 733–741. doi: 10.1523/JNEUROSCI.07-03-00733.1987, PMID: 3559710 PMC6569053

[ref97] Maison S. F. Liu X. P. Vetter D. E. Eatock R. A. Nathanson N. M. Wess J.. (2010). Muscarinic signaling in the cochlea: presynaptic and postsynaptic effects on efferent feedback and afferent excitability. J. Neurosci. 30, 6751–6762. doi: 10.1523/JNEUROSCI.5080-09.2010, PMID: 20463237 PMC3332094

[ref98] Malmierca M. S. Merchán M. A. (2004). “CHAPTER 31 - auditory system” in The rat nervous system. ed. Paxinos G.. 3rd ed (Burlington: Academic Press), 997–1082.

[ref99] Manzoor N. F. Chen G. Kaltenbach J. A. (2013). Suppression of noise-induced hyperactivity in the dorsal cochlear nucleus following application of the cholinergic agonist, carbachol. Brain Res. 1523, 28–36. doi: 10.1016/j.brainres.2013.05.025, PMID: 23721928 PMC3748938

[ref100] Martin M. R. (1981). Acetylcholinesterase-positive fibers and cell bodies in the cochlear nuclei of normal and reeler mutant mice. J. Comp. Neurol. 197, 153–167. doi: 10.1002/cne.901970112, PMID: 7229123

[ref101] McKenna T. M. Ashe J. H. Hui G. K. Weinberger N. M. (1988). Muscarinic agonists modulate spontaneous and evoked unit discharge in auditory cortex of cat. Synapse 2, 54–68. doi: 10.1002/syn.890020109, PMID: 3420531

[ref102] McKenna T. M. Ashe J. H. Weinberger N. M. (1989). Cholinergic modulation of frequency receptive fields in auditory cortex: I Frequency-specific effects of muscarinic agonists. Synapse 4, 30–43. doi: 10.1002/syn.890040105, PMID: 2672402

[ref103] Mellott J. G. Beebe N. L. Schofield B. R. (2019). Bilateral projections to the thalamus from individual neurons in the inferior colliculus. J. Comp. Neurol. 527, 1118–1126. doi: 10.1002/cne.24600, PMID: 30536721 PMC6368862

[ref104] Mellott J. G. Motts S. D. Schofield B. R. (2011). Multiple origins of cholinergic innervation of the cochlear nucleus. Neuroscience 180, 138–147. doi: 10.1016/j.neuroscience.2011.02.010, PMID: 21320579 PMC3070814

[ref105] Metherate R. (2004). Nicotinic acetylcholine receptors in sensory cortex. Learn. Mem. 11, 50–59. doi: 10.1101/lm.69904, PMID: 14747517

[ref106] Metherate R. (2011). Functional connectivity and cholinergic modulation in auditory cortex. Neurosci. Biobehav. Rev. 35, 2058–2063. doi: 10.1016/j.neubiorev.2010.11.010, PMID: 21144860 PMC3139107

[ref107] Metherate R. Ashe J. H. Weinberger N. M. (1990). Acetylcholine modifies neuronal acoustic rate-level functions in guinea pig auditory cortex by an action at muscarinic receptors. Synapse 6, 364–368. doi: 10.1002/syn.890060409, PMID: 2287993

[ref108] Metherate R. Cox C. L. Ashe J. H. (1992). Cellular bases of neocortical activation: modulation of neural oscillations by the nucleus basalis and endogenous acetylcholine. J. Neurosci. 12, 4701–4711. doi: 10.1523/JNEUROSCI.12-12-04701.1992, PMID: 1361197 PMC6575759

[ref109] Metherate R. Hsieh C. Y. (2004). Synaptic mechanisms and cholinergic regulation in auditory cortex. Prog. Brain Res. 145, 143–156. doi: 10.1016/S0079-6123(03)45010-3 14650913

[ref110] Metherate R. Intskirveli I. Kawai H. D. (2012). Nicotinic filtering of sensory processing in auditory cortex. Front. Behav. Neurosci. 6:44. doi: 10.3389/fnbeh.2012.00044 22833720 PMC3400128

[ref111] Metherate R. Weinberger N. M. (1989). Acetylcholine produces stimulus-specific receptive field alterations in cat auditory cortex. Brain Res. 480, 372–377. doi: 10.1016/0006-8993(89)90210-2, PMID: 2713663

[ref112] Metherate R. Weinberger N. M. (1990). Cholinergic modulation of responses to single tones produces tone-specific receptive field alterations in cat auditory cortex. Synapse 6, 133–145. doi: 10.1002/syn.890060204, PMID: 2237776

[ref113] Mizukawa K. McGeer P. L. Tago H. Peng J. H. McGeer E. G. Kimura H. (1986). The cholinergic system of the human hindbrain studied by choline acetyltransferase immunohistochemistry and acetylcholinesterase histochemistry. Brain Res. 379, 39–55. doi: 10.1016/0006-8993(86)90253-2 2427162

[ref114] Mooney D. M. Zhang L. Basile C. Senatorov V. V. Ngsee J. Omar A.. (2004). Distinct forms of cholinergic modulation in parallel thalamic sensory pathways. Proc. Natl. Acad. Sci. U. S. A. 101, 320–324. doi: 10.1073/pnas.0304445101, PMID: 14691260 PMC314183

[ref115] Moore J. K. Osen K. K. (1979). The cochlear nuclei in man. Am. J. Anat. 154, 393–417. doi: 10.1002/aja.1001540306 433789

[ref116] Morley B. J. Happe H. K. (2000). Cholinergic receptors: dual roles in transduction and plasticity. Hear. Res. 147, 104–112. doi: 10.1016/S0378-5955(00)00124-6, PMID: 10962177

[ref117] Morley B. J. Lorden J. F. Brown G. B. Kemp G. E. Bradley R. J. (1977). Regional distribution of nicotinic acetylcholine receptor in rat brain. Brain Res. 134, 161–166. doi: 10.1016/0006-8993(77)90935-0 912415

[ref118] Motts S. D. Slusarczyk A. S. Sowick C. S. Schofield B. R. (2008). Distribution of cholinergic cells in guinea pig brainstem. Neuroscience 154, 186–195. doi: 10.1016/j.neuroscience.2007.12.017, PMID: 18222049 PMC2475650

[ref119] Noftz W. A. Beebe N. L. Mellott J. G. Schofield B. R. (2020). Cholinergic projections from the Pedunculopontine tegmental nucleus contact excitatory and inhibitory neurons in the inferior colliculus. Front. Neural. Circuits 14:43. doi: 10.3389/fncir.2020.00043, PMID: 32765226 PMC7378781

[ref120] Oertel D. Cao X.-J. (2020). “2.27 - the ventral Cochlear nucleus” in The senses: A comprehensive reference. ed. Fritzsch B.. 2nd ed (Oxford: Elsevier), 517–532.

[ref121] Oertel D. Fay R. R. Popper A. N. (2002). Integrative functions in the mammalian auditory pathway. Springer Nature.

[ref122] Oertel D. Wu S. H. Garb M. W. Dizack C. (1990). Morphology and physiology of cells in slice preparations of the posteroventral cochlear nucleus of mice. J. Comp. Neurol. 295, 136–154. doi: 10.1002/cne.902950112, PMID: 2341631

[ref123] Oliver D. L. Beckius G. E. Shneiderman A. (1995). Axonal projections from the lateral and medial superior olive to the inferior colliculus of the cat: a study using electron microscopic autoradiography. J. Comp. Neurol. 360, 17–32. doi: 10.1002/cne.903600103, PMID: 7499562

[ref124] Oliver D. L. Hall W. C. (1978). The medial geniculate body of the tree shrew, *Tupaia glis*. II. Connections with the neocortex. J. Comp. Neurol. 182, 459–493. doi: 10.1002/cne.901820306, PMID: 102661

[ref125] Oliver D. Klocker N. Schuck J. Baukrowitz T. Ruppersberg J. P. Fakler B. (2000). Gating of Ca2+−activated K+ channels controls fast inhibitory synaptic transmission at auditory outer hair cells. Neuron 26, 595–601. doi: 10.1016/S0896-6273(00)81197-6, PMID: 10896156

[ref126] Osen K. K. (1969a). Cytoarchitecture of the cochlear nuclei in the cat. J. Comp. Neurol. 136, 453–483. doi: 10.1002/cne.901360407, PMID: 5801446

[ref127] Osen K. K. (1969b). The intrinsic organization of the cochlear nuclei. Acta Otolaryngol. 67, 352–359. doi: 10.3109/00016486909125462 5374653

[ref128] Osen K. K. (1970). Course and termination of the primary afferents in the cochlear nuclei of the cat. An experimental anatomical study. Arch. Ital. Biol. 108, 21–51, PMID: 5438720

[ref129] Osen K. K. Roth K. (1969). Histochemical localization of cholinesterases in the cochlear nuclei of the cat, with notes on the origin of acetylcholinesterase-positive afferents and the superior olive. Brain Res. 16, 165–185. doi: 10.1016/0006-8993(69)90092-4, PMID: 5348847

[ref130] Pollak G. D. Burger R. M. Klug A. (2003). Dissecting the circuitry of the auditory system. Trends Neurosci. 26, 33–39. doi: 10.1016/S0166-2236(02)00009-7, PMID: 12495861

[ref131] Pollak G. D. Wenstrup J. J. Fuzessey Z. M. (1986). Auditory processing in the mustache bat's inferior colliculus. Trends Neurosci. 9, 556–561. doi: 10.1016/0166-2236(86)90176-1

[ref132] Rasmussen G. (1960). Efferent fibers of the cochlear nerve and cochlear nucleus. Neural Mechanisms Auditory Vestibular Systems 8, 105–115.

[ref133] Rasmussen G. (1965). Efferent connections of the cochlear nucleus. Sensori Neural Hearing Processes Disord., 61–75.

[ref134] Reese N. B. Garcia-Rill E. Skinner R. D. (1995a). Auditory input to the pedunculopontine nucleus: II Unit responses. Brain Res Bull 37, 265–273. doi: 10.1016/0361-9230(95)00001-U, PMID: 7627569

[ref135] Reese N. B. Garcia-Rill E. Skinner R. D. (1995b). The pedunculopontine nucleus--auditory input, arousal and pathophysiology. Prog. Neurobiol. 47, 105–133. doi: 10.1016/0301-0082(95)00023-O, PMID: 8711130

[ref136] Rhode W. S. Oertel D. Smith P. H. (1983). Physiological response properties of cells labeled intracellularly with horseradish peroxidase in cat ventral cochlear nucleus. J. Comp. Neurol. 213, 448–463. doi: 10.1002/cne.902130408, PMID: 6300200

[ref137] Richardson B. D. Sottile S. Y. Caspary D. M. (2021). Mechanisms of GABAergic and cholinergic neurotransmission in auditory thalamus: impact of aging. Hear. Res. 402:108003. doi: 10.1016/j.heares.2020.108003, PMID: 32703637 PMC9075165

[ref138] Risoud M. Hanson J. N. Gauvrit F. Renard C. Lemesre P. E. Bonne N. X.. (2018). Sound source localization. Eur. Ann. Otorhinolaryngol. Head Neck Dis. 135, 259–264. doi: 10.1016/j.anorl.2018.04.009 29731298

[ref139] Rivera-Perez L. M. Kwapiszewski J. T. Roberts M. T. (2021). alpha3beta4 (*) nicotinic acetylcholine receptors strongly modulate the excitability of VIP neurons in the mouse inferior colliculus. Front. Neural Circuits 15:709387. doi: 10.3389/fncir.2021.709387, PMID: 34434092 PMC8381226

[ref140] Roeren T. LeVeen R. F. Nugent L. (1988). Photoplethysmographic documentation of improved microcirculation after pentoxifylline therapy. Angiology 39, 929–933. doi: 10.1177/000331978803901101, PMID: 3177959

[ref141] Romero G. E. Trussell L. O. (2022). Central circuitry and function of the cochlear efferent systems. Hear. Res. 425:108516. doi: 10.1016/j.heares.2022.108516 35606211

[ref142] Rotter A. Birdsall N. J. Field P. M. Raisman G. (1979). Muscarinic receptors in the central nervous system of the rat. II. Distribution of binding of [3H]propylbenzilylcholine mustard in the midbrain and hindbrain. Brain Res. 180, 167–183. doi: 10.1016/0165-0173(79)90003-1, PMID: 519515

[ref143] Rouiller E. M. de Ribaupierre F. (1985). Origin of afferents to physiologically defined regions of the medial geniculate body of the cat: ventral and dorsal divisions. Hear. Res. 19, 97–114. doi: 10.1016/0378-5955(85)90114-5, PMID: 4055537

[ref144] Roux I. Wersinger E. McIntosh J. M. Fuchs P. A. Glowatzki E. (2011). Onset of cholinergic efferent synaptic function in sensory hair cells of the rat cochlea. J. Neurosci. 31, 15092–15101. doi: 10.1523/JNEUROSCI.2743-11.2011, PMID: 22016543 PMC3213862

[ref145] Safieddine S. Bartolami S. Wenthold R. J. Eybalin M. (1996). Pre- and postsynaptic M3 muscarinic receptor mRNAs in the rodent peripheral auditory system. Brain Res. Mol. Brain Res. 40, 127–135, PMID: 8840020 10.1016/0169-328x(96)00047-2

[ref146] Schofield B. R. (1991). Superior paraolivary nucleus in the pigmented guinea pig: separate classes of neurons project to the inferior colliculus and the cochlear nucleus. J. Comp. Neurol. 312, 68–76. doi: 10.1002/cne.903120106, PMID: 1744244

[ref147] Schofield B. R. (2002). Ascending and descending projections from the superior olivary complex in guinea pigs: different cells project to the cochlear nucleus and the inferior colliculus. J. Comp. Neurol. 453, 217–225. doi: 10.1002/cne.10402, PMID: 12378584

[ref148] Schofield B. Hurley L. (2018). Circuits for modulation of auditory function. The mammalian auditory pathways: Synaptic organization and microcircuits. 235–267. doi: 10.1007/978-3-319-71798-2_9

[ref149] Schofield B. R. Motts S. D. Mellott J. G. (2011). Cholinergic cells of the pontomesencephalic tegmentum: connections with auditory structures from cochlear nucleus to cortex. Hear. Res. 279, 85–95. doi: 10.1016/j.heares.2010.12.019, PMID: 21195150 PMC3087857

[ref150] Servent D. Blanchet G. Mourier G. Marquer C. Marcon E. Fruchart-Gaillard C. (2011). Muscarinic toxins. Toxicon 58, 455–463. doi: 10.1016/j.toxicon.2011.08.004 21906611

[ref151] Shen J. X. Yakel J. L. (2009). Nicotinic acetylcholine receptor-mediated calcium signaling in the nervous system. Acta Pharmacol. Sin. 30, 673–680. doi: 10.1038/aps.2009.64, PMID: 19448647 PMC4002362

[ref152] Sherriff F. E. Henderson Z. (1994). Cholinergic neurons in the ventral trapezoid nucleus project to the cochlear nuclei in the rat. Neuroscience 58, 627–633. doi: 10.1016/0306-4522(94)90086-8, PMID: 7513389

[ref153] Shute C. C. Lewis P. R. (1967). The ascending cholinergic reticular system: neocortical, olfactory and subcortical projections. Brain 90, 497–520. doi: 10.1093/brain/90.3.497, PMID: 6058140

[ref154] Sottile S. Y. Ling L. Cox B. C. Caspary D. M. (2017). Impact of ageing on postsynaptic neuronal nicotinic neurotransmission in auditory thalamus. J. Physiol. 595, 5375–5385. doi: 10.1113/JP274467, PMID: 28585699 PMC5538226

[ref155] Stornetta R. L. Macon C. J. Nguyen T. M. Coates M. B. Guyenet P. G. (2013). Cholinergic neurons in the mouse rostral ventrolateral medulla target sensory afferent areas. Brain Struct. Funct. 218, 455–475. doi: 10.1007/s00429-012-0408-3, PMID: 22460939 PMC3459297

[ref156] Stotler W. A. (1953). An experimental study of the cells and connections of the superior olivary complex of the cat. J. Comp. Neurol. 98, 401–431. doi: 10.1002/cne.900980303, PMID: 13069629

[ref157] Strutz J. (1987). Anatomy of the central auditory pathway. Demonstration with horseradish peroxidase in the guinea pig. HNO 35, 407–415, PMID: 3679895

[ref158] Tachibana M. Rothman J. M. Guth P. S. (1979). Somatostatin along the auditory pathway. Hear. Res. 1, 365–368. doi: 10.1016/0378-5955(79)90006-6, PMID: 44284

[ref159] Taranda J. Maison S. F. Ballestero J. A. Katz E. Savino J. Vetter D. E.. (2009). A point mutation in the hair cell nicotinic cholinergic receptor prolongs cochlear inhibition and enhances noise protection. PLoS Biol. 7:e18. doi: 10.1371/journal.pbio.1000018 19166271 PMC2628405

[ref160] Thompson A. M. Schofield B. R. (2000). Afferent projections of the superior olivary complex. Microsc. Res. Tech. 51, 330–354. doi: 10.1002/1097-0029(20001115)51:4<330::AID-JEMT4>3.0.CO;2-X 11071718

[ref161] Uchimura N. North R. A. (1990). Muscarine reduces inwardly rectifying potassium conductance in rat nucleus accumbens neurones. J. Physiol. 422, 369–380. doi: 10.1113/jphysiol.1990.sp017989, PMID: 1693682 PMC1190137

[ref162] Uteshev V. V. (2012). alpha7 nicotinic ACh receptors as a ligand-gated source of ca(2+) ions: the search for a ca(2+) optimum. Adv. Exp. Med. Biol. 740, 603–638. doi: 10.1007/978-94-007-2888-2_27, PMID: 22453962 PMC3584641

[ref163] Varela C. Sherman S. M. (2007). Differences in response to muscarinic activation between first and higher order thalamic relays. J. Neurophysiol. 98, 3538–3547. doi: 10.1152/jn.00578.2007, PMID: 17942627

[ref164] Wang H. S. Pan Z. Shi W. Brown B. S. Wymore R. S. Cohen I. S.. (1998). KCNQ2 and KCNQ3 potassium channel subunits: molecular correlates of the M-channel. Science 282, 1890–1893. doi: 10.1126/science.282.5395.1890, PMID: 9836639

[ref165] Watanabe T. Simada Z. (1973). Pharmacological properties of cat's collicular auditory neurons. Jpn. J. Physiol. 23, 291–308. doi: 10.2170/jjphysiol.23.291, PMID: 4270927

[ref166] Weber M. Motin L. Gaul S. Beker F. Fink R. H. Adams D. J. (2005). Intravenous anaesthetics inhibit nicotinic acetylcholine receptor-mediated currents and Ca2+ transients in rat intracardiac ganglion neurons. Br. J. Pharmacol. 144, 98–107. doi: 10.1038/sj.bjp.0705942, PMID: 15644873 PMC1575970

[ref167] Weimann S. R. Zhang C. Burger R. M. (2024). A developmental switch in cholinergic mechanisms of modulation in the medial nucleus of the trapezoid body. J. Neurosci. 44:e0356232023. doi: 10.1523/JNEUROSCI.0356-23.2023 38383485 PMC10883614

[ref168] Winslow R. L. Sachs M. B. (1988). Single-tone intensity discrimination based on auditory-nerve rate responses in backgrounds of quiet, noise, and with stimulation of the crossed olivocochlear bundle. Hear. Res. 35, 165–189. doi: 10.1016/0378-5955(88)90116-5, PMID: 3198509

[ref169] Wolpert S. Heyd A. Wagner W. (2014). Assessment of the noise-protective action of the olivocochlear efferents in humans. Audiol. Neurootol. 19, 31–40. doi: 10.1159/000354913, PMID: 24281009

[ref170] Womble M. D. Moises H. C. (1992). Muscarinic inhibition of M-current and a potassium leak conductance in neurones of the rat basolateral amygdala. J. Physiol. 457, 93–114. doi: 10.1113/jphysiol.1992.sp019366, PMID: 1338469 PMC1175719

[ref171] Yao W. Godfrey D. A. (1995). Immunohistochemistry of muscarinic acetylcholine receptors in rat cochlear nucleus. Hear. Res. 89, 76–85. doi: 10.1016/0378-5955(95)00123-7, PMID: 8600134

[ref172] Yao W. Godfrey D. A. (1996). Autoradiographic distribution of muscarinic acetylcholine receptor subtypes in rat Cochlear nucleus. Audit. Neurosci. 2, 241–255, PMID: 33510562 PMC7840062

[ref173] Yin T. C. Chan J. C. (1990). Interaural time sensitivity in medial superior olive of cat. J. Neurophysiol. 64, 465–488. doi: 10.1152/jn.1990.64.2.465, PMID: 2213127

[ref174] Young E. D. Brownell W. E. (1976). Responses to tones and noise of single cells in dorsal cochlear nucleus of unanesthetized cats. J. Neurophysiol. 39, 282–300. doi: 10.1152/jn.1976.39.2.282, PMID: 1255224

[ref175] Zhang C. Beebe N. L. Schofield B. R. Pecka M. Burger R. M. (2021). Endogenous cholinergic signaling modulates sound-evoked responses of the medial nucleus of the trapezoid body. J. Neurosci. 41, 674–688. doi: 10.1523/JNEUROSCI.1633-20.2020, PMID: 33268542 PMC7842756

[ref176] Zhao Y. Tzounopoulos T. (2011). Physiological activation of cholinergic inputs controls associative synaptic plasticity via modulation of endocannabinoid signaling. J. Neurosci. 31, 3158–3168. doi: 10.1523/JNEUROSCI.5303-10.2011, PMID: 21368027 PMC3111389

[ref177] Zook J. M. Casseday J. H. (1987). Convergence of ascending pathways at the inferior colliculus of the mustache bat, *Pteronotus parnellii*. J. Comp. Neurol. 261, 347–361. doi: 10.1002/cne.902610303, PMID: 3611416

